# Supplementary material for: Regulatory standards and guidance for the use of health applications for self-management in Africa: scoping review protocol
Source: BMJ Open. 2022 Feb 11;12(2):e058067. doi: 10.1136/bmjopen-2021-058067 (PMC8845322; doi:10.1136/bmjopen-2021-058067)
Supplement: Supplementary data [file bmjopen-2021-058067supp002.pdf]

Supplementary file 1: Sample search strategy for Scopus

Database(s): **Scopus** 1960 to October 2021 (final search limited to 2005 – current)

Search Strategy:

ID	Query	Documents
#6	((polic* OR legislat* OR strateg* OR regulat* OR standard* OR criter* OR framework* OR guideline*)) AND (digital AND health OR ehealth OR e-health OR mhealth OR m-health) AND ((application AND software OR app* OR software AND app* OR mobile AND app* OR mobile AND phone AND app* OR medical AND app*)) AND ((world AND health AND organization OR who AND african AND region OR africa OR sub-saharan AND africa OR "Africa South of the Sahara") OR (algeria OR angola OR benin OR botswana OR burkina AND faso OR burundi OR cameroon OR cape AND verde OR cabo AND verde OR central AND african AND republic OR chad OR comoros OR ivory AND coast OR cote AND d'ivoire OR democratic AND republic AND of AND the AND congo OR equatorial AND guinea OR eritrea OR ethiopia OR gabon OR gambia OR ghana OR guinea OR guinea-bissau OR kenya OR lesotho OR liberia OR madagascar OR malawi OR mali OR mauritania OR mauritius OR mozambique OR namibia OR niger OR nigeria OR republic AND of AND the AND congo OR congo OR rwanda OR são AND tomé AND príncipe OR saint AND thomas AND prince OR senegal OR seychelles OR sierra AND leone OR south AND africa OR south AND sudan OR eswatini OR togo OR uganda OR tanzania OR zambia OR zimbabwe)) AND (LIMIT-TO (PUBYEAR , 2021) OR LIMIT-TO (PUBYEAR , 2020) OR LIMIT-TO (PUBYEAR , 2019) OR LIMIT-TO (PUBYEAR , 2018) OR LIMIT-TO (PUBYEAR , 2017) OR LIMIT-TO (PUBYEAR , 2016) OR LIMIT-TO (PUBYEAR , 2015) OR LIMIT-TO (PUBYEAR , 2014) OR LIMIT-TO (PUBYEAR , 2013) OR LIMIT-TO (PUBYEAR , 2012) OR LIMIT-TO (PUBYEAR , 2011) OR LIMIT-TO (PUBYEAR , 2010) OR LIMIT-TO (PUBYEAR , 2009) OR LIMIT-TO (PUBYEAR , 2008) OR LIMIT-TO (PUBYEAR , 2007) OR LIMIT-TO (PUBYEAR , 2006) OR LIMIT-TO (PUBYEAR , 2005))	1,054
#5	((polic* OR legislat* OR strateg* OR regulat* OR standard* OR criter* OR framework* OR guideline*)) AND (digital AND health OR ehealth OR e-health OR mhealth OR m-health) AND ((application AND software OR app* OR software AND app* OR mobile AND app* OR mobile AND phone AND app*	1,057

	OR medical AND app*)) AND ((world AND health AND organization OR who AND african AND region OR africa OR sub-saharan AND africa OR "Africa South of the Sahara") OR (algeria OR angola OR benin OR botswana OR burkina AND faso OR burundi OR cameroon OR cape AND verde OR cabo AND verde OR central AND african AND republic OR chad OR comoros OR ivory AND coast OR cote AND d'ivoire OR democratic AND republic AND of AND the AND congo OR equatorial AND guinea OR eritrea OR ethiopia OR gabon OR gambia OR ghana OR guinea OR guinea-bissau OR kenya OR lesotho OR liberia OR madagascar OR malawi OR mali OR mauritania OR mauritius OR mozambique OR namibia OR niger OR nigeria OR republic AND of AND the AND congo OR congo OR rwanda OR são AND tomé AND príncipe OR saint AND thomas AND prince OR senegal OR seychelles OR sierra AND leone OR south AND africa OR south AND sudan OR eswatini OR togo OR uganda OR tanzania OR zambia OR zimbabwe))	
#4	(world AND health AND organization OR who AND african AND region OR africa OR sub-saharan AND africa OR "Africa South of the Sahara") OR (algeria OR angola OR benin OR botswana OR burkina AND faso OR burundi OR cameroon OR cape AND verde OR cabo AND verde OR central AND african AND republic OR chad OR comoros OR ivory AND coast OR cote AND d'ivoire OR democratic AND republic AND of AND the AND congo OR equatorial AND guinea OR eritrea OR ethiopia OR gabon OR gambia OR ghana OR guinea OR guinea-bissau OR kenya OR lesotho OR liberia OR madagascar OR malawi OR mali OR mauritania OR mauritius OR mozambique OR namibia OR niger OR nigeria OR republic AND of AND the AND congo OR congo OR rwanda OR são AND tomé AND príncipe OR saint AND thomas AND prince OR senegal OR seychelles OR sierra AND leone OR south AND africa OR south AND sudan OR eswatini OR togo OR uganda OR tanzania OR zambia OR zimbabwe)	143,892
#3	(application AND software OR app* OR software AND app* OR mobile AND app* OR mobile AND phone AND app* OR medical AND app*)	208,315
#2	digital AND health OR ehealth OR e-health OR mhealth OR m-health	495,739
#1	(polic* OR legislat* OR strateg* OR regulat* OR standard* OR criter* OR framework* OR guideline*)	30,603,666
