# Supplementary material for: Regulatory standards and guidance for the use of health applications for self-management in Africa: scoping review protocol
Source: BMJ Open. 2022 Feb 11;12(2):e058067. doi: 10.1136/bmjopen-2021-058067 (PMC8845322; doi:10.1136/bmjopen-2021-058067)
Supplement: Author's manuscript [file bmjopen-2021-058067.draft_revisions.pdf]

BMJ Open

BMJ Open is committed to open peer review. As part of this commitment we make the peer review history of every article we publish publicly available.

When an article is published we post the peer reviewers' comments and the authors' responses online. We also post the versions of the paper that were used during peer review. These are the versions that the peer review comments apply to.

The versions of the paper that follow are the versions that were submitted during the peer review process. They are not the versions of record or the final published versions. They should not be cited or distributed as the published version of this manuscript.

BMJ Open is an open access journal and the full, final, typeset and author-corrected version of record of the manuscript is available on our site with no access controls, subscription charges or pay-per-view fees (http://bmjopen.bmj.com).

If you have any questions on BMJ Open's open peer review process please email info.bmjopen@bmj.com

BMJ Open

Regulatory Standards and Guidance for the Use of Health Apps for Self-Management in Africa: Scoping Review Protocol

Journal:	*BMJ Open*
Manuscript ID	bmjopen-2021-058067
Article Type:	Protocol
Date Submitted by the Author:	15-Oct-2021
Complete List of Authors:	Bene, Benard; Imperial College London School of Public Health, Department of Primary Care & Public Health; Federal Ministry of Health, Department of Public Health<br>Mastellos , Nikolaos; Imperial College London,<br>Majeed, Azeem; Imperial College London, Primary Care<br>Fadahunsi, Kayode; Imperial College London, Primary Care and Public Health<br>Ibeneme, Sunny ; World Health Organization – African Regional Office, Brazzaville, Congo<br>Harri, Bala; Federal Ministry of Health, Department of Health Planning, Research and Statistics<br>Ukor, Nkiruka ; World Health Organization – Nigeria Country Office, Abuja<br>Car, Josip; Imperial College London, Primary Care and Social Medicine
Keywords:	Health policy < HEALTH SERVICES ADMINISTRATION & MANAGEMENT, Protocols & guidelines < HEALTH SERVICES ADMINISTRATION & MANAGEMENT, Quality in health care < HEALTH SERVICES ADMINISTRATION & MANAGEMENT, Clinical governance < HEALTH SERVICES ADMINISTRATION & MANAGEMENT, Information technology < BIOTECHNOLOGY & BIOINFORMATICS

SCHOLARONE™
Manuscripts

Regulatory Standards and Guidance for the Use of Health Apps for Self-Management in Africa: Scoping Review Protocol

Benard Ayaka Bene[1,2], Nikolaos Mastellos[1], Azeem Majeed[1], Kayode Philip Fadahunsi[1], Sunny Ibeneme[3], Bala Isah Harri[4], Nkiruka Ukor[5], Josip Car[1*]

[1] Department of Primary Care and Public Health, Imperial College London, United Kingdom.

[2] Department of Public Health, Federal Ministry of Health, Abuja, Nigeria.

[3] Department of Health Planning, Research and Statistics at the Federal Ministry of Health, Nigeria.

[4] World Health Organization – African Regional Office, Brazzaville, Congo.

[5] World Health Organization – Nigeria Country Office, Abuja.

***Corresponding Author**

Josip Car

Department of Primary Care and Public Health, Imperial College London, United Kingdom.

josip.car@imperial.ac.uk

Key words: Regulations, standards, guidance, health apps, self-management, WHO African Region, sub-Saharan Africa

Word count: 4664

For peer review only

Abstract

Introduction

Despite health apps becoming ubiquitous and with enormous potential to facilitate self-management, key issues such as app quality, data privacy, and interoperability are barriers to their adoption. While some high-income countries have made significant strides in establishing standards for regulation of health apps and their adoption by health systems, it is unclear what is the progress in Africa. This scoping review protocol describes steps we will take to investigate the extent to which regulatory standards and guidance address the use of health apps for self-management within the WHO African Region countries.

Methods

The review will follow the 6-stage methodological framework for conducting scoping reviews described by Arksey and O'Malley (2005) (1). The following electronic databases will be searched from inception to October 2021: Ovid Medline, Embase, Global Health, CINAHL, and Scopus. Google, OPENGREY, WHO AFROLIB, African Index Medicus (AIM). Websites of relevant institutions, and repositories for digital health policies will also be searched. Additional documents will be identified by searching the reference lists of included documents and by contacting key persons at the Ministries of Health or other relevant institutions/departments or the WHO Country Office or the WHO Regional Office for Africa. Results obtained will be reported using descriptive qualitative content analysis based on the review objectives. The policy analysis framework by Walt and Gilson (1994) (2) will be used to organise findings regarding the health apps for self-management. A summary of the key findings will be presented using tables, charts, and maps.

Ethics and dissemination

The collection of primary data is not anticipated in this study and hence ethical approval will not be required. The review will be published in a peer-reviewed journal while key findings will be shared with relevant organisations and/or presented at conferences.

For peer review only

Strengths and limitations of this study

- The study will identify standards and guidance for regulating the use of health apps for self-management within the WHO African Region countries.
- We will compare the regulatory standards and guidance that regulate the use of health apps for self-management in terms of what aspects are regulated, why, how and for whom, and what is not regulated in Africa.
- The study will identify gaps in the health app regulatory environment which will inform the design of future research
- The study will also map out the key actors and their roles in regulating the use of health apps for patient self-management in Africa.
- Regulatory standards or national policies on digital health which are not published may not be publicly available. However, efforts will be made to obtain these documents by contacting key persons at the Ministries of Health and/or other relevant institutions/departments, the WHO Country Office in respective countries, or the WHO Regional Office for Africa.

For peer review only

Background

Evolution of digital health

Digital health has been defined as the application or utilisation of information and communications technology (ICT) within the health domain (3). Digital health has advanced remarkably over the past decade from electronic health (eHealth) concepts including mobile health (mHealth), telemedicine, and electronic health records (EHRs) to the application of more advanced computing sciences such as big data, blockchain, and artificial intelligence (3–6). This has created new opportunities for health systems to become more efficient and responsive to people's needs, with the potential to advance Universal Health Coverage (UHC) and accelerate the attainment of Goal 3 of the Sustainable Development Goals (SDG), which is good health and wellbeing (3,7–9).

Owing to the recent COVID-19 pandemic, digital health has further gained momentum in areas such as remote consultations, remote monitoring of vital signs, and self-care (10–14). Consequently, there is renewed interest from governments, investors, technology companies, health practitioners, patients, the general population, and other relevant stakeholders to explore the potentials of digital health (6,15,16). As these potentials of digital health are being explored, the landscape of digital health continues to expand with more advances in intervention areas such as digital self-care, digital therapeutics, as well as creating new evidence-based knowledge, skills, and competence for key players in the health and social care sector (6,9,17).

Emergence of digital self-care

Digital self-care, an emerging area of digital health, is the intersection between the use of digital technologies (particularly, mobile health solutions) and self-care (17). While self-care is the broader term used to describe a range of interventions including self-awareness, self-testing, and self-management (17,18), this study will focus on self-management. Self-management, which is considered a crucial component of chronic disease management, incorporates the daily

For peer review only

practices that individuals intentionally carry out to maintain good health as guided by their health care providers (19,20).

The world is experiencing massive proliferation of mobile apps, better internet access, and advances in artificial intelligence, thus making it increasingly easier for people to engage in self-management (17,21–23). A recent report indicated that 218 billion mobile apps were downloaded in 2020, with an estimated global expenditure of over $240 billion in app stores (23).

A mobile app, which is a software application designed to run on mobile devices (such as smartphones and tablet computers), can provide an important platform or tool for self-management and can be used both as a delivery mechanism and a self-management intervention (17,24). The use of apps for self-management has potential to increase access to and reach (coverage) of effective and safe healthcare services at scale whilst also promoting autonomy, person-centred care, privacy and confidentiality as well as promoting equity, efficient use of resources (especially in Africa) and other low-and middle-income countries (LMICs) where resources are grossly inadequate (17,25–28). More specifically, mobile apps designed for health purposes are commonly referred to as health apps (29–32). These apps have the unique ability to facilitate one or more aspects of self-management of diseases by capturing user's health data and providing tailored information, instructions, graphic displays, guidance, and reminders as well as enabling remote monitoring of patient's health status whilst also linking patients to their healthcare providers and social networks (24). Rather than manually recording user's health data, some health apps are designed to allow user's data such as blood glucose, blood pressure, heart rate, and weight to be automatically transmitted from measuring devices, wearables, or sensors (via Bluetooth) (33).

While the use of self-management health apps presents an opportunity to strengthen health systems, the potential of these apps has hardly been harnessed in Africa and other low-and middle-income countries (LMICs). For over a decade, mHealth-related interventions in Africa

For peer review only

have been restricted to services that do not require smartphones or internet connection, such as short messaging service (SMS) and use of voice notes (17,34,35). This is partly because of the limited access to smartphones and low penetration of internet services on the continent. However, this narrative is changing as there is increased availability of cheaper smartphones, and the cost of mobile internet subscription is rapidly declining in Africa (36), thus presenting an immense opportunity to close the digital heath divide that had existed between Africa and high-income countries for decades. According to the GSM Association, the adoption of smartphones in sub-Saharan Africa continues to rise rapidly moving from 10% of total smartphone connections in 2014 to about 50% in 2020 (37,38). Mobile network operators in Africa will therefore continue to upgrade their services in the coming years and hence narrow the gap between 2G and 3G coverage (38). In addition, the introduction of new smartphone financing models, such as the partnership between Safaricom and Google in Kenya, is making it increasingly possible for individuals of low economic status to afford 4G devices through daily instalments (37). It is further estimated that there will be about 678 million smartphone connections in sub-Sharan Africa by the end of 2025 with the adoption rate increasing to 65% (37). By implication, the number of Africans who can use health apps for self-management is expected to increase substantially over the next few years.

Although self-management health apps present many opportunities and potential for the continent of Africa, appropriate regulatory standards and guidance are required to facilitate adoption of evidence-based, safe and efficient (amongst others) health apps (33,39). While this is a huge challenge for high-income countries thus impeding the maturity of health apps as a fully integrated component of the healthcare system, Africa has a golden opportunity of addressing this challenge early. This will allow Africa to leapfrog some of the barriers that many high-income countries have had to deal with because of the delay in establishing appropriate and well-coordinated regulatory standards.

For peer review only

Key regulatory issues related the use of heath apps for self-management and the imperative for appropriate regulatory standards and guidance

There have been several fragmented efforts in developing frameworks for assessment of health apps' quality (including trustworthiness, effectiveness, and safety) (40–46). However, there is so far no global consensus on standards and guidance that wholistically regulate health apps for self-management. This has contributed greatly to the proliferation of low-quality health apps which can be harmful to users (38,45).

While high-income countries such as the United Kingdom (UK) and the United States of America (USA) as well as the continent of Europe have made significant progress in establishing standardised processes for evaluating (including rating and classifying), certifying and selecting health apps (32,48–51), it is unclear to what extent countries in Africa have progressed in establishing standards that address regulatory issues related the use of heath apps for self-management.

The number of health apps on app stores now exceeds 300,000, and yet there is very limited empirical evidence of app effectiveness and safety (52). Up to 85% of health apps reviewed by ORCHA (the Organisation for the Review of Care and Health Applications) did not meet expected quality threshold (53). A study by Huckvale and colleagues found that widely used health apps which calculated and estimated insulin doses posed a danger to patients by giving incorrect or inappropriate dose recommendations (47). Similarly, two successive studies that assessed the contents and tools of apps for asthma discovered that none of the apps in the first study offered comprehensive information or adequate tools for asthma self-management while the follow-up study, which was conducted two years later, showed a 2-fold increase in the number of asthma apps, yet there was no improvement to the content and tools offered by the newer apps. In fact, many of the apps recommended self-management procedures that were not supported by evidence (54,55). These findings suggest that not only are health apps for self-management of long-term conditions poorly designed (do not adhere to evidence-based

For peer review only

guidelines and offer limited clinical benefits) but are unresponsive to the evolving health needs of patients.

Furthermore, some health apps are associated with poor compliance to ethical standards and the principles of confidentiality of information and data privacy. This could erode the trust and confidence of patients. An assessment of apps that had been endorsed by the UK National Health Service (NHS) Apps Library revealed significant gaps in compliance with data protection principles regarding collection, storage, and transmission of personal information. This has raised a fundamental concern about the credibility of developer disclosures and whether these disclosures can be trusted by certification programs (56). A more recent study assessed privacy practices of 36 most popular apps for depression and smoking cessation for Android and iOS in the United States and Australia (57). The findings revealed that though only 25 of 36 apps (69%) included privacy policy, 33 of all 36 apps (92%) shared data with a third party and only 23 (among the 25 with privacy policy) disclosed sharing data with a third party in their policy. While 29 of the 36 apps (81%) shared data with Google and Facebook for the purposes of advertising, marketing or analytics, only 12 of 28 that shared data with Google and 6 of 12 that shared data with Facebook disclosed this in their policy (57). These findings reinforce the need for more robust accreditation programs that will ensure that before health apps are released, publishers explicitly indicate how they intend to manage or use personal information and give patients the options to accept or decline any request to share their personal information with a third party. In addition, publishers must resolve violation risks to privacy policy before publishing health apps, while users are consistently warned against potential security threats and violation risk to their personal information (56,57).

Also, limited interoperability both within and across healthcare organisations is making it difficult to get health apps to interact with other health IT systems (33,39). This is so because most health apps are developed outside of healthcare systems, hence the potential benefits that can be derived from apps being a part of an overall integrated system are not maximised. For instance, it is impossible to remotely monitor patient's symptoms or health status from a health

For peer review only - http://bmjopen.bmj.com/site/about/guidelines.xhtml

app or link the patient to healthcare providers for real-time support without first integrating the app to EHR or within the clinical workflows (33). Therefore, there is a need for national standards for interoperability to be developed and enforced from the start while local and regional efforts are made to promote interoperability and data sharing (16,58).

Global efforts toward promoting the appropriate use of digital technologies as digital public goods

The Word Health Organization (WHO), cognisant of the role of digital health in shaping the future of global health, has since 2005 made several efforts to encourage and support countries especially in Africa to establish regulatory standards to minimise variability and potential harms that may arise from poorly regulated use of digital health (9,59). At the fifty-eighth World Health Assembly (WHA) in 2005, resolution WHA58.28 on eHealth was passed and adopted by Member States (60). Subsequently, in 2013, resolution WHA66.24 on eHealth standardisation and interoperability was adopted at the Sixty-Sixth WHA (61). And more recently, in 2018, resolution WHA71.7 on digital health was adopted at the Seventy-First WHA by Member States (62). Consolidating on these key resolutions, WHO has further developed implementation frameworks to provide guidance and support to member states to develop and implement national policies, strategies, and regulatory standard for digital health. These implementation frameworks include the National eHealth Strategy Toolkit (in collaboration with International Telecommunication Union [ITU]) (7); WHO Guideline: Recommendations on Digital Interventions for Health System Strengthening (63); Digital Health Platform Handbook: Building a Digital Information Infrastructure (Infostructure) for Health (in collaboration with the ITU) (16); Digital Implementation Investment Guide (DIIG): Integrating Digital Interventions into Health Programmes (64); Ethics and Governance of Artificial Intelligence for Health: WHO Guidance (65); and WHO guidance and Global Strategy on Digital Health 2020–2025 (which builds on United Nations General Assembly resolutions 73/218 and 70/125) (9).

For peer review only

Following the efforts of the WHO and other key stakeholders, many countries including those in Africa now have some form of national policy or strategy on digital health (9). However, beyond having national policies and strategies on digital health, it is imperative for countries to have regulatory standards and guidance that specifically address key regulatory issues related to the use of health apps based on country-specific contexts. So far, it is unclear which countries in the WHO African Region have these regulatory standards. The Global Digital Health Index and Maturity Model, an initiative of HealthEnabled and its partners, was launched in 2018 to serve as an interactive digital platform for tracking, monitoring, and evaluating the use of digital health technologies across different countries (66,67). The inaugural report on the Global Digital Health Index was subsequently published in 2019 (66). To the best of our knowledge, this inaugural report is the first attempt to evaluate countries' progress in digital health using 19 core indicators grouped under seven categories of key indicators of the WHO/ITU's National eHealth Strategy Toolkit (leadership and governance; strategy and investment; legislation, policy, and compliance; workforce; standards and interoperability; infrastructure; and services and applications). Although the report provided valuable insights into the current state of legislation, policy, and compliance as well as standards and interoperability across countries, only five of 47 countries (Benin, Ethiopia, Mali, Nigeria, Sierra Leone, and Uganda) in the WHO African Region participated in the evaluation. Moreover, the report did not provide any insight on whether countries have standalone regulatory standards and guidance on digital health and whether existing policies and strategies address key issues related to the use of health apps for self-management.

In this paper, we present a protocol for a scoping review to investigate the extent to which regulatory standards and guidance address the aforementioned key regulatory issues related to the use of health apps for self-management in countries[1] within the WHO African Region.

---

[1] Algeria, Angola, Benin, Botswana, Burkina Faso, Burundi, Cameroon, Cape Verde, Central African Republic, Chad, Comoros, Ivory Coast, Democratic Republic of the Congo, Equatorial Guinea, Eritrea, Ethiopia, Gabon, Gambia, Ghana, Guinea, Guinea-Bissau, Kenya, Lesotho, Liberia, Madagascar, Malawi, Mali, Mauritania, Mauritius, Mozambique, Namibia, Niger, Nigeria, Republic of the Congo, Rwanda, São Tomé and Príncipe, Senegal, Seychelles, Sierra Leone, South Africa, South Sudan, Eswatini, Togo, Uganda, Tanzania, Zambia, Zimbabwe.

For peer review only - http://bmjopen.bmj.com/site/about/guidelines.xhtml

Aim and Objectives

Aim

To identify and compare the extent to which regulatory standards and guidance regulate the use of health apps for self-management, and to map out the key actors and their responsibilities in regulating the use of health apps for patient self-management within the WHO African Region countries.

Objectives

The objectives of this review are to:

1. *Identify* regulatory standards and guidance that are available for regulating the use of health apps for self-management within the WHO African Region countries.
2. *Compare* the extent to which the regulatory standards and guidance regulate the use of health apps for self-management in terms of what aspects are regulated, why, how and for whom, and what aspects are not regulated.
3. *Map* out the key actors and their roles in regulating the use of health apps for patient self-management.

Methods

Study design

A team comprising experts and researchers with experience and interest in the relevant disciplines (digital health, self-management, health policy and planning, and scoping review methodology) will design, conduct and report the scoping review. The process of the scoping review will follow the 6-stage methodological framework for conducting a scoping study which was originally described by Arksey and O'Malley (2005) and subsequently modified by Anderson et al. (2008) and Levac et al. (2010), and most recently by Peters et al. (2020) (1,68–70). While stage 1 of the framework will guide the formation of the review question and search strategy, stage 2 and stage 3 will subsequently guide the identification of relevant studies and the study selection process respectively. Stage 4 will guide the development of the data-

For peer review only

charting form as well as the data charting (extraction) process itself, whereas stage 5 will guide the process of collation, summarisation, and reporting of results. The final stage (stage 6) is optional and will guide the process of consultation with actors (if required). The reporting of the scoping review will be guided by the Preferred Reporting Items for Systematic Reviews and Meta-Analyses extension for Scoping Reviews (PRISMA-ScR) checklist (71). The PRISMA-ScR checklist will be completed and attached as an additional file.

This review is not registered with PROSPERO or any similar registration platform as it is not a requirement for scoping reviews to be pre-registered (70).

Stage 1: Identifying the research questions

Research questions

The review will attempt to answer the following research questions:

1. What regulatory standards and guidance are available for regulating the use of health apps for self-management within the WHO African Region countries?
2. To what extent do regulatory standards and guidance regulate the use of health apps for self-management in terms of what aspects are regulated, why, how and for whom, and what aspects are not regulated?
3. Who are the key actors and what are their roles in regulating the use of health apps for patient self-management?

Criteria for considering sources of evidence for this review

Inclusion criteria

The sources of evidence that will be considered for inclusion in this review are:

- Standalone regulatory standards, guidance, and committee or government reports that potentially address regulatory issues related to the use of health apps in any WHO African Region country.
- National policies and strategies on digital health developed by countries within the WHO African Region.

For peer review only

- Sources of evidence developed, written, or published between 2005 and September 2021. This is because it was in 2005 that global efforts toward encouraging and supporting countries to establish regulatory standards to minimise variability and potential harms that could arise from poorly regulated use of digital health began (9).

Exclusion criteria

- Policies, strategies, regulatory standards, guidance, and committee or government reports that address the use of health apps for patient self-management in countries outside the WHO African Region.

Stage 2: Identifying relevant studies

A comprehensive search strategy will be designed by two reviewers with the assistance of a librarian and in consultation with other research team members. The following key terms will be included: policies, legislations, strategies, regulations, standards, criteria, frameworks, guidelines, digital health, eHealth, apps, WHO African Region, and sub-Saharan Africa, and list of all countries within the WHO African Region. We will also use truncation to increase yield of results.

The search strategy will then be used to search for all eligible policy related sources of evidence including conference proceedings and grey literature (committee reports and government reports). Boolean terms (mainly 'OR' and 'AND') will be used to combine search results. 'OR' will be used to combine results within the same category while 'AND' will be used to combine result between different categories to narrow down the search results.

The following electronic databases will be searched from inception to October 2021: Ovid Medline; Embase; Global Health; CINAHL; and Scopus. To locate unpublished evidence and reports, we will search Google; OPENGREY; WHO Regional Office for Africa Library (AFROLIB); African Index Medicus (AIM); and websites of relevant institutions (including ITU, WHO, Ministries of Health of all the countries within the WHO African Region). Repositories for digital health policies (including ICT Works, WHO directory of eHealth policies; and HIS Strengthening Resource Centre) will also be searched. Additional relevant

For peer review only

documents will be identified by searching the reference lists of included sources of evidence or by contacting key persons at the Ministries of Health or other relevant institutions/departments or the WHO Country Office in respective countries as well as the WHO Regional Office for Africa.

A sample search strategy for Ovid Medline is provided in supplementary file 1.

Stage 3: Study selection

Sources of evidence identified from searches and key contact persons will be imported into Mendeley reference management software to remove duplicates. The remaining sources of evidence will subsequently be imported into Covidence (a web-based tool that allows two reviewers to independently manage article screening and data extraction). Using the predefined eligibility criteria outlined in Stage 2, two reviewers, working independently, will screen each source of evidence in two stages (title and abstract, and full text) for possible inclusion in the review. Any discrepancies will be discussed in order to reach a consensus. Unresolved disagreements will be further deliberated on and resolved in a steering group meeting involving a third reviewer.

The entire selection processes will be guided by the Preferred Reporting Items for Systematic Reviews and Meta-Analyses extension for Scoping Reviews (PRISMA-ScR) checklist (71).

Stage 4: Charting (extraction) the data

Two reviewers (BAB and KPF) in consultation with the other members of the research team will develop the data extraction form using an iterative process. At the initial stage of this process, a pilot data extraction will be carried out on at least 10 percent of included sources of evidence. The form will continuously be refined until a consensus is reached.

Following the development of the data extraction form, full data extraction will then be done by the two reviewers independently and any disagreement will be resolved by discussion. Inconsistencies or unresolved issues will be discussed and resolved with a third reviewer (JC)

For peer review only

in a steering group meeting. Any missing information that is relevant to this review will be sought from the actors/relevant stakeholders during the consultation stage.

As recommended by Peters et al (70), the data to be extracted will include author (s); year of publication or production; country of origin (where the source was published or produced); type of document (e.g. standalone regulatory standards on digital health or mHealth or health apps, and national policy or strategy on digital health); document development approach (e.g. public-private/multisectoral approach or private sector approach), the aspects of the use of health apps for self-management that are regulated, the reasons why they are regulated, how the regulations are enforced, for which population are the regulations targeted such as apps users, app developers and healthcare providers, and the aspects of the use of health apps for self-management that are not regulated; and the key actors and their responsibilities in regulating the use of health apps for patient self-management.

Stage 5: Collating, summarizing, and reporting the results

Unlike systematic reviews, the goal of scoping reviews is not to synthesise the results or outcomes of included sources of evidence; however, as recommended by Peters et al, the scoping review authors may choose to extract results and descriptively (rather than analytically) map them (70). Therefore, data extracted in stage 4 will be reported using descriptive qualitative content analysis based on the review objectives and as an attempt answer the research questions. The policy analysis framework by Walt and Gilson (1994) (2) will be used to organise findings regarding the aspects of the use of health apps for self-management that are regulated, the reasons why they are regulated, how the regulations are enforced, for which population are the regulations targeted. This will be done based on four priori categories, namely: context, content, process, and actors. The Reporting Items for Stakeholder Analysis (i.e., the RISA tool) (72) will be used to guide the mapping of key actors and their responsibilities in regulating the use of health apps for patient self-management.

Summary of the key findings will be presented using tables, charts, and geospatial map(s). Charts and maps are appropriate for aiding the visualisation of the key findings (62). More

For peer review only

specifically, key actors and their responsibilities in regulating the use of health apps for patient self-management will be summarised in a map.

Stage 6: Consultation

Consultation with policy actors or other relevant stakeholders will only be considered in this review if additional information is needed to better understand the policy process, involvement of actors, as well as contextual factors (including social, economic, and political) regarding the sources of evidence examined. Once the purpose of a consultation is established, application for ethical approval will be made and then the policy actors or stakeholders will be contacted for an interview following ethical approval. However, the data collected is likely to be analysed and reported as part of a separate study.

Patient and Public Involvement

There was no direct involvement of patients and the public in the design of this study. However, there is a plan to consult with stakeholders (including patients) if additional information is required.

Ethics and Dissemination

This study is mainly a review of regulatory standards and guidance. Collection of primary data through consultation with stakeholders will be done only if necessary. Application for ethical approval will be made to the Imperial College Research Ethics Committee (ICRC) through the Head of Primary Care and Public Health Department, Imperial College London if required.

On completion of the study, a manuscript will be submitted to a peer-reviewed journal for publication while summary of the findings will be shared with relevant organisations. Key findings will be summarised and presented at national, regional, and international conferences.

Conclusion

To ensure that citizens of countries within the WHO African Region have access to safe and effective health apps for self-management, and to promote full adoption of health apps by the

For peer review only

healthcare system, governments must establish regulatory standards and guidance that will address key aspects of the use health apps for self-management.

The need for regulatory standards and guidance for the use of health apps for self-management cannot be overemphasised considering that health apps are becoming ubiquitous, and they potentially provide an effective tool for facilitating one or more aspects of self-management. In addition, there is a growing body of evidence suggesting that quality assurance processes for health apps are not well-coordinated and are unresponsive to evolving health needs whilst also not complying to ethical standards and principles of confidentiality of information and data privacy. There is also an acute need for national standards for interoperability to be established at the early stages of digitalisation of the healthcare system. This allows health apps and other digital health interventions to interact with each other as well as with the health IT system.

While several individual efforts have been made in the past to develop frameworks for assessing the quality of health apps, there has not been a global consensus on standards and guidance that wholistically regulate health apps for self-management. The USA, and the continent of Europe as well as some European countries such as the UK have made good progress in establishing standards and guidance which potentially address key regulatory issues related the use of heath apps for self-management. It is however unclear if such progress has been made in Africa. Therefore, identifying and comparing the extent to which regulatory standards and guidance regulate the use of health apps for self-management, and mapping out the key actors and their responsibilities in regulating the use of health apps for patient self-management within the WHO African Region countries will help identify existing gaps in the health app regulatory environment. This will subsequently inform the design of future research as well as inform service design for the use of health apps for self-management in countries within the WHO African Region.

For peer review only

This study is part of a series of studies which together form the first attempt at exploring the digital readiness of the health system of a LMIC in Africa to integrate health apps for self-management of type 2 diabetes.

Acknowledgments

We appreciate Rebecca Jones, the Library Manager and Liaison Librarian at the Charing Cross Library, Imperial College London, for her advice and assistance with the search strategy for the main review.

Footnotes

Contributors

BAB and JC conceived the study. BAB designed the study with contributions from JC and NM. BAB drafted the manuscript and JC, NM, AM, KPF, SI, BIH, and NU read and contributed to it. AM is the clinical lead while JC acts as guarantor for the study. The final manuscript was read and approved by all the authors.

Funding

This work was supported by NIHR Applied Research Collaboration North-West London, grant number NIHR200180. The views expressed in this publication are those of the authors and not necessarily those of the NIHR or the Department of Health and Social Care.

Competing interests

None declared.

Patient consent for publication

Not required

For peer review only

References

1. Arksey H, O'Malley L. Scoping studies: Towards a methodological framework. International Journal of Social Research Methodology: Theory and Practice. 2005 Feb;8(1):19–32.

2. Walt G, Gilson L. Reforming the health sector in developing countries: the central role of policy analysis [Internet]. Vol. 9, HEALTH POLICY AND PLANNING. 1994. Available from: https://academic.oup.com/heapol/article/9/4/353/649125

3. WHO. WHO guideline: Recommendations on digital interventions for health system strengthening. Geneva; 2019.

4. FDA. What is Digital Health? [Internet]. 2020 [cited 2021 Jun 30]. Available from: https://www.fda.gov/medical-devices/digital-health-center-excellence/what-digital-health

5. Palandrani P, Little A. A Decade of Change: How Tech Evolved in the 2010's and What's In Store for the 2020's [Internet]. Global X. 2020 [cited 2021 Jul 5]. Available from: https://www.globalxetfs.com/a-decade-of-change-how-tech-evolved-in-the-2010s-and-whats-in-store-for-the-2020s/

6. Dang A, Arora D, Rane P. Role of digital therapeutics and the changing future of healthcare. Journal of Family Medicine and Primary Care. 2020;9(5):2207.

7. WHO, ITU. National eHealth Strategy Toolkit [Internet]. 2012 [cited 2021 Jun 28]. Available from: https://www.itu.int/pub/D-STR-E_HEALTH.05-2012

8. Broadband Commission. Digital Health: A Call for Government Leadership and Cooperation between ICT and Health - Broadband Commission [Internet]. 2017 [cited 2021 Jun 28]. Available from: https://www.broadbandcommission.org/publication/digital-health/

9. WHO. Global strategy on digital health 2020-2025 [Internet]. 2021 [cited 2021 Jun 23]. Available from: http://apps.who.int/bookorders.

10. Gupta SK, Lakshmi PVM, Kaur M, Rastogi A. Role of self-care in COVID-19 pandemic for people living with comorbidities of diabetes and hypertension. Journal of Family Medicine and Primary Care [Internet]. 2020 [cited 2021 Aug 5];9(11):5495. Available from: /pmc/articles/PMC7842493/

11. Gunasekeran D v, Tham Y-C, Ting DSW, Tan GSW, Wong TY. Digital health during COVID-19: lessons from operationalising new models of care in ophthalmology. The Lancet Digital Health [Internet]. 2021 Feb 1 [cited 2021 Aug 5];3(2):e124–34. Available from: http://www.thelancet.com/article/S2589750020302879/fulltext

12. Norton M. How COVID-19 is highlighting the importance of digital health solutions [Internet]. 2020 [cited 2021 Aug 5]. Available from: https://www.med-technews.com/medtech-insights/how-covid-19-is-highlighting-the-importance-of-digital-healt/

13. Budd J, Miller BS, Manning EM, Lampos V, Zhuang M, Edelstein M, et al. Digital technologies in the public-health response to COVID-19. Nature Medicine 2020 26:8 [Internet]. 2020 Aug 7 [cited 2021 Aug 5];26(8):1183–92. Available from: https://www.nature.com/articles/s41591-020-1011-4

14. Murphy M, Scott LJ, Salisbury C, Turner A, Scott A, Denholm R, et al. Implementation of remote consulting in UK primary care following the COVID-19 pandemic: a mixed-methods longitudinal study. British Journal of General Practice [Internet]. 2021 Mar 1 [cited 2021 Aug 5];71(704):e166–77. Available from: https://bjgp.org/content/71/704/e166

For peer review only

15. Runway Innovation Hub. Digital Health: Categories & Trends [Internet]. 2020 [cited 2021 Jun 30]. Available from: https://runway.is/blog/digital-health-trends/

16. WHO, ITU. Digital Health Platform Handbook: Building a Digital Information Infrastructure (Infostructure) for Health. Geneva; 2020.

17. Chaney SC, Mechael P, Self-Care Trailblazer Group. Digital Self Care: A Framework for Design, Implementation & Evaluation [Internet]. 2020 [cited 2021 May 20]. Available from: https://www.psi.org/wp-content/uploads/2020/09/Digital-Self-Care.pdf

18. El-Osta A, Webber D, Gnani S, Banarsee R, Mummery D, Majeed A, et al. The Self-Care Matrix: A unifying framework for self-care. Selfcare Journal [Internet]. 2019 [cited 2021 Sep 29];10(3):38–56. Available from: https://selfcarejournal.com/article/the-self-care-matrix-a-unifying-framework-for-self-care/

19. Lorig KR, Holman HR. Self-management education: History, definition, outcomes, and mechanisms. Annals of Behavioral Medicine [Internet]. 2003 Aug 1 [cited 2021 Sep 29];26(1):1–7. Available from: https://academic.oup.com/abm/article/26/1/1/4630312

20. Barlow J, Wright C, Sheasby J, Turner A, Hainsworth J. Self-management approaches for people with chronic conditions: a review. Patient Education and Counseling. 2002 Oct 1;48(2):177–87.

21. Aikens JE, Zivin K, Trivedi R, Piette JD. Diabetes self-management support using mHealth and enhanced informal caregiving. Journal of Diabetes and its Complications. 2014;28(2):171–6.

22. Jeffrey B, Bagala M, Creighton A, Leavey T, Nicholls S, Wood C, et al. Mobile phone applications and their use in the self-management of Type 2 Diabetes Mellitus: a qualitative study among app users and non-app users. Diabetol Metab Syndr [Internet]. 2019 [cited 2021 May 31];11:84. Available from: https://doi.org/10.1186/s13098-019-0480-4

23. RiskIQ. Mobile App Threat Landscape Report [Internet]. 2020 [cited 2021 Jul 19]. Available from: https://www.riskiq.com/2020-mobile-threat-landscape-report-thank-you/

24. Bene BA, O'Connor S, Mastellos N, Majeed A, Fadahunsi KP, O'Donoghue J. Impact of mobile health applications on self-management in patients with type 2 diabetes mellitus: protocol of a systematic review. BMJ Open [Internet]. 2019 Jun 1 [cited 2021 Jul 19];9(6):e025714. Available from: https://bmjopen.bmj.com/content/9/6/e025714

25. Olu O, Muneene D, Bataringaya JE, Nahimana M-R, Ba H, Turgeon Y, et al. How Can Digital Health Technologies Contribute to Sustainable Attainment of Universal Health Coverage in Africa? A Perspective. Frontiers in Public Health [Internet]. 2019 Nov 15 [cited 2021 Mar 21];7:341. Available from: https://www.frontiersin.org/article/10.3389/fpubh.2019.00341/full

26. Wanless D. Securing our Future Health: Taking a Long-Term View Final Report. 2002.

27. WHO. The MAPS Toolkit: mHealth Assessment and Planning for Scale [Internet]. Geneva; 2015 [cited 2021 Mar 15]. Available from: www.who.int

28. Wallis L, Blessing P, Dalwai M, Shin S do. Integrating mHealth at point of care in low- and middle-income settings: the system perspective. Global Health Action [Internet]. 2017 [cited 2021 Jul 18];10(sup3). Available from: /pmc/articles/PMC5645717/

29. Peng W, Kanthawala S, Yuan S, Hussain SA. A qualitative study of user perceptions of mobile health apps. BMC Public Health [Internet]. 2016 Nov 14 [cited 2021 Sep 20];16(1):1–11. Available from: https://bmcpublichealth.biomedcentral.com/articles/10.1186/s12889-016-3808-0

For peer review only

30. Krebs P, Duncan DT. Health App Use Among US Mobile Phone Owners: A National Survey. JMIR mHealth and uHealth [Internet]. 2015 Nov 4 [cited 2021 Sep 20];3(4):e4924. Available from: https://mhealth.jmir.org/2015/4/e101

31. Anderson K, Burford O, Emmerton L. Mobile Health Apps to Facilitate Self-Care: A Qualitative Study of User Experiences. PLOS ONE [Internet]. 2016 May 1 [cited 2021 Sep 20];11(5):e0156164. Available from: https://journals.plos.org/plosone/article?id=10.1371/journal.pone.0156164

32. Gordon WJ, Landman A, Zhang H, Bates DW. Beyond validation: getting health apps into clinical practice. npj Digital Medicine 2020 3:1 [Internet]. 2020 Feb 3 [cited 2021 Sep 20];3(1):1–6. Available from: https://www.nature.com/articles/s41746-019-0212-z

33. Aitken M, Lyle J. Patient Adoption of mHealth: Use, Evidence and Remaining Barriers to Mainstream Acceptance [Internet]. 2015 [cited 2021 May 21]. Available from: www.theimsinstitute.org

34. Piette JD, Lun KC, Moura LA, Fraser HS, Mechael PN, Powell J, et al. Policy & practice Impacts of e-health on the outcomes of care in low-and middle-income countries: where do we go from here? Bull World Health Organ. 2012;90:365–72.

35. Mechael P, Batavia H, Kaonga N, Searle S, Kwan A, Goldberger A, et al. Barriers and Gaps Affecting mHealth in Low and Middle Income Countries: Policy White Paper [Internet]. Columbia; 2010. Available from: http://www.globalproblems-globalsolutions-files.org/pdfs/mHealth_Barriers_White_Paper.pdf

36. World Health Organisation. Global diffusion of eHealth: Making universal health coverage achievable [Internet]. Geneva; 2016 [cited 2021 Jul 18]. Available from: http://apps.who.int/bookorders.

37. GSM Association. The Mobile Economy Sub-Saharan Africa 2020 [Internet]. 2020 [cited 2021 Sep 28]. Available from: https://www.gsma.com/mobileeconomy/wp-content/uploads/2020/09/GSMA_MobileEconomy2020_SSA_Eng.pdf

38. Bahia K, Suardi S. The State of Mobile Internet Connectivity 2019 [Internet]. 2019 [cited 2021 Sep 27]. Available from: https://www.gsma.com/mobilefordevelopment/wp-content/uploads/2019/07/GSMA-State-of-Mobile-Internet-Connectivity-Report-2019.pdf

39. Aitken M, Gauntlett C. Patient Apps for Improved Healthcare: From Novelty to Mainstream [Internet]. IMS Institute for Healthcare Informatics. Parsippany, NJ; 2013. Available from: http://www.imshealth.com/portal/site/imshealth

40. Torous J, Chan S, Gipson S-M, al. et. A hierarchical framework for evaluation and informed decision making regarding smartphone apps for clinical care. Psychiatr Serv. 2018;69:498–500.

41. Moshi M, Tooher R, Merlin T. Suitability of current evaluation frameworks for use in the health technology assessment of mobile medical applications: a systematic review. Int J Technol Assess Health Care. 2018;34:464–75.

42. Henson P, David G, Albright K, Torous J. Deriving a practical framework for the evaluation of health apps. The Lancet Digital Health [Internet]. 2019 Jun 1 [cited 2021 Jul 30];1(2):e52–4. Available from: http://www.thelancet.com/article/S2589750019300135/fulltext

43. Lagan S, Sandler L, Torous J. Evaluating evaluation frameworks: A scoping review of frameworks for assessing health apps. BMJ Open. 2021 Mar 19;11(3).

For peer review only

44. Hensher M, Cooper P, Dona SWA, Angeles MR, Nguyen D, Heynsbergh N, et al. Scoping review: Development and assessment of evaluation frameworks of mobile health apps for recommendations to consumers. Journal of the American Medical Informatics Association : JAMIA [Internet]. 2021 Jun 12 [cited 2021 Jul 30];28(6):1318–29. Available from: https://pubmed.ncbi.nlm.nih.gov/33787894/

45. Guan Z, Sun L, Xiao Q, Wang Y. Constructing an assessment framework for the quality of asthma smartphone applications. BMC Medical Informatics and Decision Making 2019 19:1 [Internet]. 2019 Oct 15 [cited 2021 Jul 30];19(1):1–17. Available from: https://bmcmedinformdecismak.biomedcentral.com/articles/10.1186/s12911-019-0923-8

46. Stoyanov SR, Hides L, Kavanagh DJ, Zelenko O, Tjondronegoro D, Mani M. Mobile App Rating Scale: A New Tool for Assessing the Quality of Health Mobile Apps. JMIR Mhealth Uhealth [Internet]. 2015 Mar 11 [cited 2021 Aug 5];3(1):e3422. Available from: https://mhealth.jmir.org/2015/1/e27

47. Huckvale K, Adomaviciute S, Prieto JT, Leow MK, Car J. Smartphone apps for calculating insulin dose: a systematic assessment. BMC Medicine [Internet]. 2015;13(1):106. Available from: http://www.ncbi.nlm.nih.gov/pubmed/25943590

48. Joyce R, Harkins L. Streamlining digital health technology assessments [Internet]. 2020 [cited 2021 Jul 28]. Available from: https://www.nhsx.nhs.uk/blogs/dtac/

49. Unsworth H, Dillon B, Collinson L, Powell H, Salmon M, Oladapo T, et al. The NICE Evidence Standards Framework for digital health and care technologies – Developing and maintaining an innovative evidence framework with global impact: https://doi.org/101177/20552076211018617 [Internet]. 2021 Jun 24 [cited 2021 Jul 28];7:205520762110186. Available from: https://journals.sagepub.com/doi/full/10.1177/20552076211018617

50. FDA. Device Software Functions Including Mobile Medical Applications | FDA [Internet]. 2019 [cited 2021 Jul 28]. Available from: https://www.fda.gov/medical-devices/digital-health-center-excellence/device-software-functions-including-mobile-medical-applications

51. Medicines & Healthcare Products Regulatory Agency. Guidance: Medical device stand-alone software including apps (including IVDMDs) . 2021.

52. Bene BA, O'Connor S, Mastellos N, Majeed A, Fadahunsi KP, O'Donoghue J. Impact of mobile health applications on self-management in patients with type 2 diabetes mellitus: Protocol of a systematic review. BMJ Open. 2019;9(6).

53. ORCHA. Coronavirus: Apps to help the elderly [Internet]. 2020 [cited 2021 Jul 19]. Available from: https://orchahealth.com/coronavirus-apps-to-help-the-elderly/

54. Huckvale K, Car M, Morrison C, Car J. Apps for asthma self-management: a systematic assessment of content and tools. BMC medicine [Internet]. 2012;10(1):144. Available from: http://www.biomedcentral.com/1741-7015/10/144

55. Huckvale K, Morrison C, Ouyang J, Ghaghda A, Car J. The evolution of mobile apps for asthma: an updated systematic assessment of content and tools. BMC Medicine [Internet]. 2015;13(1):58. Available from: http://www.ncbi.nlm.nih.gov/pubmed/25857569

56. Huckvale K, Prieto JT, Tilney M, Benghozi P-J, Car J. Unaddressed privacy risks in accredited health and wellness apps: a cross-sectional systematic assessment. BMC Medicine 2015 13:1 [Internet]. 2015 Sep 25 [cited 2021 Jul 21];13(1):1–13. Available from: https://bmcmedicine.biomedcentral.com/articles/10.1186/s12916-015-0444-y

For peer review only

57. Huckvale K, Torous J, Larsen ME. Assessment of the Data Sharing and Privacy Practices of Smartphone Apps for Depression and Smoking Cessation. JAMA Network Open [Internet]. 2019 Apr 5 [cited 2021 Jul 21];2(4):e192542–e192542. Available from: https://jamanetwork.com/journals/jamanetworkopen/fullarticle/2730782

58. Wachter RM. Making IT Work: Harnessing the Power of Health Information Technology to Improve Care in England [Internet]. 2016 [cited 2021 Jul 22]. Available from: https://assets.publishing.service.gov.uk/government/uploads/system/uploads/attachment_data/file/550866/Wachter_Review_Accessible.pdf

59. Ibeneme S, Ukor N, Ongom M, Dasa T, Muneene D, Okeibunor J. Strengthening capacities among digital health leaders for the development and implementation of national digital health programs in Nigeria. In: BMC Proceedings [Internet]. BioMed Central; 2020 [cited 2021 May 23]. p. 1–12. Available from: https://doi.org/10.1186/s12919-020-00193-1

60. WHO. The 58th World Health Assembly (WHA58.28) on eHealth. 2005.

61. WHO. SIXTY-SIXTH WORLD HEALTH ASSEMBLY. Geneva; 2013.

62. WHO. Seventy-first World Health Assembly [Internet]. 2018 [cited 2021 Jul 1]. Available from: https://apps.who.int/gb/ebwha/pdf_files/WHA71-REC1/A71_2018_REC1-en.pdf#page=25

63. WHO. WHO Guideline: recommendations on digital interventions for health system strengthening [Internet]. WHO. World Health Organization; 2019 [cited 2021 May 21]. Available from: http://www.who.int/reproductivehealth/publications/digital-interventions-health-system-strengthening/en/

64. World Health Organisation. Digital Implementation Investment Guide (DIIG): Integrating Digital Interventions into Health Programmes [Internet]. 2020 [cited 2021 Sep 28]. Available from: https://www.who.int/publications/i/item/9789240010567

65. World Health Organisation. Ethics and governance of artificial intelligence for health: WHO guidance [Internet]. World Health Organization. Geneva: WHO; 2021 [cited 2021 Sep 28]. Available from: http://apps.who.int/bookorders.

66. Mechael P, Edelman JK. Global Digital Health Index: The State of Digital Health 2019. 2019.

67. Global Digital Health Index [Internet]. [cited 2021 Jul 29]. Available from: https://www.digitalhealthindex.org/

68. Anderson S, Allen P, Peckham S, Goodwin N. Asking the right questions: Scoping studies in the commissioning of research on the organisation and delivery of health services. Vol. 6, Health Research Policy and Systems. 2008.

69. Levac D, Colquhoun H, O'Brien KK. Scoping studies: Advancing the methodology. Implementation Science. 2010 Sep 20;5(1).

70. Peters M, Godfrey C, McInerney P, Munn Z, Trico A, Khalil H. Chapter 11: Scoping Reviews (2020 version). In: Aromataris E, Munn Z, editors. JBI Manual for Evidence Synthesis [Internet]. JBI; 2020 [cited 2021 Aug 23]. Available from: https://synthesismanual.jbi.global. https://doi.org/10.46658/JBIMES-20-12

71. Tricco AC, Lillie E, Zarin W, O'Brien KK, Colquhoun H, Levac D, et al. PRISMA Extension for Scoping Reviews (PRISMA-ScR): Checklist and Explanation. https://doi.org/107326/M18-0850 [Internet]. 2018 Sep 4 [cited 2021 Jul 30];169(7):467–73. Available from: https://www.acpjournals.org/doi/abs/10.7326/M18-0850

72. Franco-Trigo L, Fernandez-Llimos F, Martínez-Martínez F, Benrimoj SI, Sabater-Hernández D. Stakeholder analysis in health innovation planning processes: A systematic scoping review.

For peer review only - http://bmjopen.bmj.com/site/about/guidelines.xhtml

Health Policy [Internet]. 2020 [cited 2021 Sep 3];124:1083–99. Available from: https://doi.org/10.1016/j.healthpol.2020.06.012

73. Wariri O, Okomo U, Kwarshak YK, Murray KA, Grundy C, Kampmann B. Timeliness of routine childhood vaccination in low- and middle-income countries, 1978–2021: Protocol for a scoping review to map methodologic gaps and determinants. PLOS ONE [Internet]. 2021 Jun 1 [cited 2021 Aug 22];16(6):e0253423. Available from: https://journals.plos.org/plosone/article?id=10.1371/journal.pone.0253423

Supplementary file 1: Sample search strategy for Ovid Medline

Database(s): **Ovid MEDLINE(R) ALL** 1946 to September 27, 2021
Search Strategy:

#	Searches	Results
1	(Polic* or legislat* or strateg* or regulat* or standard* or criter* framework* or guideline*).mp. [mp=title, abstract, original title, name of substance word, subject heading word, floating sub-heading word, keyword heading word, organism supplementary concept word, protocol supplementary concept word, rare disease supplementary concept word, unique identifier, synonyms]	6218582
2	Digital Health.mp.	3653
3	eHealth.mp.	5083
4	e-Health.mp.	3367
5	(mHealth or m-Health).mp. [mp=title, abstract, original title, name of substance word, subject heading word, floating sub-heading word, keyword heading word, organism supplementary concept word, protocol supplementary concept word, rare disease supplementary concept word, unique identifier, synonyms]	7090
6	2 or 3 or 4 or 5	17024
7	(Application software or app* or software app* or mobile app* or mobile phone app* or medical app*).mp. [mp=title, abstract, original title, name of substance word, subject heading word, floating sub-heading word, keyword heading word, organism supplementary concept word, protocol supplementary concept word, rare disease supplementary concept word, unique identifier, synonyms]	7267195
8	World Health Organization/ or WHO African Region.mp. or Africa/	66724
9	Sub-Saharan Africa.mp. or "Africa South of the Sahara"/	31418
10	Algeria.mp. or Algeria/	4681
11	Angola.mp. or Angola/	1768
12	Benin.mp. or Benin/	4229
13	Botswana.mp. or Botswana/	2961

14	Burkina Faso.mp. or Burkina Faso/	5047
15	Burundi.mp. or Burundi/	1099
16	Cameroon.mp. or Cameroon/	8713
17	Cape Verde.mp. or Cabo Verde/	637
18	Central African Republic/ or Central African Republic.mp.	1280
19	Chad.mp. or Chad/	1453
20	Comoros.mp. or Comoros/	568
21	Ivory Coast.mp. or Cote d'Ivoire/	3967
22	Democratic Republic of the Congo.mp. or "Democratic Republic of the Congo"/	5958
23	Equatorial Guinea.mp. or Equatorial Guinea/	518
24	Eritrea.mp. or Eritrea/	720
25	Ethiopia.mp. or Ethiopia/	22717
26	Gabon.mp. or Gabon/	2264
27	Gambia.mp. or Gambia/	3375
28	Ghana.mp. or Ghana/	13782
29	Guinea.mp. or Guinea/	164230
30	Guinea-Bissau.mp. or Guinea-Bissau/	1322
31	Kenya.mp. or Kenya/	24128
32	Lesotho/ or Lesotho.mp.	845
33	Liberia.mp. or Liberia/	2046
34	Madagascar.mp. or Madagascar/	5842
35	Malawi.mp. or Malawi/	8579
36	Mali.mp. or Mali/	4391
37	Mauritania.mp. or Mauritania/	788
38	Mauritius.mp. or Mauritius/	1191
39	Mozambique.mp. or Mozambique/	4370
40	Namibia.mp. or Namibia/	2018
41	Niger/ or Niger.mp.	14716
42	Nigeria.mp. or Nigeria/	40877
43	Republic of the Congo.mp. or Congo/	7886
44	Rwanda.mp. or Rwanda/	4018
45	(Saint Thomas and Prince).mp. [mp=title, abstract, original title, name of substance word, subject heading word, floating sub-heading word, keyword heading word, organism supplementary concept word, protocol supplementary concept word, rare disease supplementary concept word, unique identifier, synonyms]	1

46	Senegal.mp. or Senegal/	8134
47	Seychelles.mp. or Seychelles/	848
48	Sierra Leone.mp. or Sierra Leone/	2799
49	South Africa.mp. or South Africa/	58715
50	South Sudan.mp. or South Sudan/	643
51	Eswatini.mp. or Eswatini/	755
52	Togo.mp. or Togo/	1868
53	Uganda.mp. or Uganda/	18633
54	Tanzania.mp. or Tanzania/	16804
55	Zambia.mp. or Zambia/	6915
56	Zimbabwe.mp. or Zimbabwe/	8228
57	8 or 9 or 10 or 11 or 12 or 13 or 14 or 15 or 16 or 17 or 18 or 19 or 20 or 21 or 22 or 23 or 24 or 25 or 26 or 27 or 28 or 29 or 30 or 31 or 32 or 33 or 34 or 35 or 36 or 37 or 38 or 39 or 40 or 41 or 42 or 43 or 44 or 45 or 46 or 47 or 48 or 49 or 50 or 51 or 52 or 53 or 54 or 55 or 56	519767
58	1 and 6 and 7 and 57	255

For peer review only

Supplementary file 1: Sample search strategy for Ovid Medline

Database(s): **Ovid MEDLINE(R) ALL** 1946 to September 27, 2021
Search Strategy:

#	Searches	Results
1	(Polic* or legislat* or strateg* or regulat* or standard* or criter* framework* or guideline*).mp. [mp=title, abstract, original title, name of substance word, subject heading word, floating sub-heading word, keyword heading word, organism supplementary concept word, protocol supplementary concept word, rare disease supplementary concept word, unique identifier, synonyms]	6218582
2	Digital Health.mp.	3653
3	eHealth.mp.	5083
4	e-Health.mp.	3367
5	(mHealth or m-Health).mp. [mp=title, abstract, original title, name of substance word, subject heading word, floating sub-heading word, keyword heading word, organism supplementary concept word, protocol supplementary concept word, rare disease supplementary concept word, unique identifier, synonyms]	7090
6	2 or 3 or 4 or 5	17024
7	(Application software or app* or software app* or mobile app* or mobile phone app* or medical app*).mp. [mp=title, abstract, original title, name of substance word, subject heading word, floating sub-heading word, keyword heading word, organism supplementary concept word, protocol supplementary concept word, rare disease supplementary concept word, unique identifier, synonyms]	7267195
8	World Health Organization/ or WHO African Region.mp. or Africa/	66724
9	Sub-Saharan Africa.mp. or "Africa South of the Sahara"/	31418
10	Algeria.mp. or Algeria/	4681
11	Angola.mp. or Angola/	1768
12	Benin.mp. or Benin/	4229
13	Botswana.mp. or Botswana/	2961
14	Burkina Faso.mp. or Burkina Faso/	5047
15	Burundi.mp. or Burundi/	1099
16	Cameroon.mp. or Cameroon/	8713
17	Cape Verde.mp. or Cabo Verde/	637
18	Central African Republic/ or Central African Republic.mp.	1280
19	Chad.mp. or Chad/	1453

20	Comoros.mp. or Comoros/	568
21	Ivory Coast.mp. or Cote d'Ivoire/	3967
22	Democratic Republic of the Congo.mp. or "Democratic Republic of the Congo"/	5958
23	Equatorial Guinea.mp. or Equatorial Guinea/	518
24	Eritrea.mp. or Eritrea/	720
25	Ethiopia.mp. or Ethiopia/	22717
26	Gabon.mp. or Gabon/	2264
27	Gambia.mp. or Gambia/	3375
28	Ghana.mp. or Ghana/	13782
29	Guinea.mp. or Guinea/	164230
30	Guinea-Bissau.mp. or Guinea-Bissau/	1322
31	Kenya.mp. or Kenya/	24128
32	Lesotho/ or Lesotho.mp.	845
33	Liberia.mp. or Liberia/	2046
34	Madagascar.mp. or Madagascar/	5842
35	Malawi.mp. or Malawi/	8579
36	Mali.mp. or Mali/	4391
37	Mauritania.mp. or Mauritania/	788
38	Mauritius.mp. or Mauritius/	1191
39	Mozambique.mp. or Mozambique/	4370
40	Namibia.mp. or Namibia/	2018
41	Niger/ or Niger.mp.	14716
42	Nigeria.mp. or Nigeria/	40877
43	Republic of the Congo.mp. or Congo/	7886
44	Rwanda.mp. or Rwanda/	4018
45	(Saint Thomas and Prince).mp. [mp=title, abstract, original title, name of substance word, subject heading word, floating sub-heading word, keyword heading word, organism supplementary concept word, protocol supplementary concept word, rare disease supplementary concept word, unique identifier, synonyms]	1
46	Senegal.mp. or Senegal/	8134
47	Seychelles.mp. or Seychelles/	848
48	Sierra Leone.mp. or Sierra Leone/	2799
49	South Africa.mp. or South Africa/	58715
50	South Sudan.mp. or South Sudan/	643
51	Eswatini.mp. or Eswatini/	755

52	Togo.mp. or Togo/	1868
53	Uganda.mp. or Uganda/	18633
54	Tanzania.mp. or Tanzania/	16804
55	Zambia.mp. or Zambia/	6915
56	Zimbabwe.mp. or Zimbabwe/	8228
57	8 or 9 or 10 or 11 or 12 or 13 or 14 or 15 or 16 or 17 or 18 or 19 or 20 or 21 or 22 or 23 or 24 or 25 or 26 or 27 or 28 or 29 or 30 or 31 or 32 or 33 or 34 or 35 or 36 or 37 or 38 or 39 or 40 or 41 or 42 or 43 or 44 or 45 or 46 or 47 or 48 or 49 or 50 or 51 or 52 or 53 or 54 or 55 or 56	519767
58	1 and 6 and 7 and 57	255

For peer review only

BMJ Open

Regulatory Standards and Guidance for the Use of Health Apps for Self-Management in Africa: Scoping Review Protocol

Journal:	*BMJ Open*
Manuscript ID	bmjopen-2021-058067.R1
Article Type:	Protocol
Date Submitted by the Author:	30-Dec-2021
Complete List of Authors:	Bene, Benard; Imperial College London School of Public Health, Department of Primary Care & Public Health; Federal Ministry of Health, Department of Public Health<br>Ibeneme, Sunny ; World Health Organization – African Regional Office, Brazzaville, Congo<br>Fadahunsi, Kayode; Imperial College London, Primary Care and Public Health<br>Harri, Bala; Federal Ministry of Health, Department of Health Planning, Research and Statistics<br>Ukor, Nkiruka ; World Health Organization – Nigeria Country Office, Abuja<br>Mastellos , Nikolaos; Imperial College London,<br>Majeed, Azeem; Imperial College London, Primary Care<br>Car, Josip; Imperial College London, Primary Care and Social Medicine
**Primary Subject Heading**:	Public health
Secondary Subject Heading:	Health policy, Health informatics, Patient-centred medicine, Health services research
Keywords:	Health policy < HEALTH SERVICES ADMINISTRATION & MANAGEMENT, Protocols & guidelines < HEALTH SERVICES ADMINISTRATION & MANAGEMENT, Quality in health care < HEALTH SERVICES ADMINISTRATION & MANAGEMENT, Clinical governance < HEALTH SERVICES ADMINISTRATION & MANAGEMENT, Information technology < BIOTECHNOLOGY & BIOINFORMATICS

SCHOLARONE™
Manuscripts

Regulatory Standards and Guidance for the Use of Health Apps for Self-Management in Africa: Scoping Review Protocol

Benard Ayaka Bene[1,2], Sunny Ibeneme[3], Kayode Philip Fadahunsi[1], Bala Isah Harri[4], Nkiruka Ukor[5], Nikolaos Mastellos[1], Azeem Majeed[1], Josip Car[1*]

[1] Department of Primary Care and Public Health, Imperial College London, United Kingdom.

[2] Department of Public Health, Federal Ministry of Health, Abuja, Nigeria.

[3] World Health Organization – African Regional Office, Brazzaville, Congo

[4] Department of Health Planning, Research and Statistics at the Federal Ministry of Health, Nigeria.

[5] World Health Organization – Nigeria Country Office, Abuja.

***Corresponding Author**

Josip Car
Department of Primary Care and Public Health, Imperial College London, United Kingdom.
josip.car@imperial.ac.uk

Key words: Regulation, standard, framework, guidance, guideline, health app, self-management, WHO African Region, sub-Saharan Africa

Word count: 2770

For peer review only

Abstract

Introduction

Despite health apps becoming ubiquitous and with enormous potential to facilitate self-management, regulatory challenges such as poor app quality, breach of data privacy, and limited interoperability have impeded their full adoption. While many countries now have digital health-related policies/strategies, there is also a need for regulatory standards and guidance that address key regulatory challenges associated with the use of health apps. Currently, it is unclear the status of countries in Africa regarding regulatory standards and guidance that address the use of health apps.

This protocol describes the process of conducting a scoping review which aims to investigate the extent to which regulatory standards and guidance address the use of health apps for self-management within the World Health Organization (WHO) African Region countries.

Methods

The review will follow the methodological framework for conducting a scoping study by Arksey and O'Malley (2005), and the updated methodological guidance for conducting a JBI scoping review. Given that regulatory standards and guidance are unlikely to be available in scientific databases, we will search Scopus, Google, OPENGREY, WHO Regional Office for Africa Library (AFROLIB), African Index Medicus (AIM), websites of WHO, ITU and Ministries of Health, repositories for digital health policies. We will also search the reference lists of included documents, and contact key stakeholders in the region. Results will be reported using descriptive qualitative content analysis based on the review objectives. The policy analysis framework by Walt and Gilson (1994) will be used to organise findings. A summary of the key findings will be presented using tables, charts, and maps.

Ethics and dissemination

The collection of primary data is not anticipated in this study and hence ethical approval will not be required. The review will be published in a peer-reviewed journal while key findings will be shared with relevant organizations and/or presented at conferences.

For peer review only - http://bmjopen.bmj.com/site/about/guidelines.xhtml

Strengths and limitations of this study

- This study, to the best of our knowledge, is the first attempt to identify gaps in the health app regulatory environment across the WHO African Region countries.
- The study will also map out the key stakeholders and their roles in regulating the use of health apps for patient self-management across the WHO African Region countries.
- Considering that regulatory standards and guidance may not be publicly available, it is possible that some documents may be missed; however, efforts will be made to obtain these documents by contacting key stakeholders.

For peer review only

For peer review only - http://bmjopen.bmj.com/site/about/guidelines.xhtml

Background

Self-management is a crucial component of chronic disease management, and it incorporates the daily practices carried out by individuals intentionally and with the guidance of their healthcare providers to maintain good health (1,2). As the world experiences a massive proliferation of mobile apps, better internet access, and advances in artificial intelligence, it is increasingly becoming easier for people to engage in self-management (3–6).

A mobile app, which is a software application designed to run on mobile devices such as smartphones and tablet computers, can provide an important platform for self-management (3,7). Health apps have the unique ability to facilitate one or more aspects of self-management of diseases by capturing the user's health data, and providing tailored information, instructions, graphic displays, guidance, and reminders (8–12). They enable remote monitoring of patient's health status whilst also linking patients to their healthcare providers and social networks (8–12). Rather than manually recording the user's health data, some health apps are designed to allow patient's health data such as blood glucose, blood pressure, heart rate, and weight to be automatically transmitted to the app (via Bluetooth) from measuring devices, wearables, or sensors (13).

The use of health apps for self-management could increase access to and reach (coverage) of effective and safe healthcare services at scale whilst also promoting autonomy, person-centred care, equity, and privacy and confidentiality (3,14–17). This also presents an opportunity to strengthen health systems in Africa and other low-and middle-income countries (LMICs) where the potential of these apps has hardly been harnessed. For over a decade, mobile health-related interventions in Africa have been restricted to services that do not require smartphones or internet connection, such as short messaging service (SMS) and use of voice notes (3,18,19). This is partly because of the limited access to smartphones and low penetration of internet services on the continent. However, this narrative is changing as there is increased availability of cheaper smartphones, and the cost of mobile internet subscription is rapidly declining (20), thus presenting an immense opportunity to close the digital heath divide that had existed between Africa and high-income countries for decades. The adoption of smartphones in sub-Saharan Africa continues to rise rapidly moving from 10% of total smartphone connections in 2014 to about 50% in 2020. This is expected to rise to 65% by the end of 2025 with about 678 million smartphone connections (21,22).

For peer review only - http://bmjopen.bmj.com/site/about/guidelines.xhtml

Because health apps for self-management present many opportunities and potential for the continent of Africa, appropriate regulatory standards and guidance are required to facilitate the adoption of evidence-based, safe, and efficient health apps (13,23). The World Health Organization (WHO) affirms that the existence of regulatory standards and guidance play an important role in strengthening digital health governance at both national and international levels (24,25). They provide a framework and guidance (with clearly defined roles) for data governance that ensures data protection, confidentiality and integrity of personal health data and system accessibility (25). Globally, the delay in establishing appropriate regulatory standards and guidance has led to the persistence of major regulatory challenges including poor app quality (26–30), breach of data privacy (31,32) and limited interoperability (13,33–35), thus impeding the maturity of health apps (as a fully integrated component of the healthcare system) (13). Africa, however, has a golden opportunity of addressing these challenges early and thus leapfrogging the barriers that many high-income countries have had to deal with (36).

Many countries including those in Africa now have some form of national policies or strategies on digital health (25). However, beyond having national policies and strategies on digital health, it is imperative for countries in Africa to have regulatory standards and guidance that address key regulatory challenges affecting the use of health apps. So far, it is unclear which countries in the WHO African Region have such regulatory standards and guidance. The Global Digital Health Index and Maturity Model, an initiative of HealthEnabled and its partners, was launched in 2018 to serve as an interactive digital platform for tracking, monitoring, and evaluating the use of digital health technologies across different countries (37,38). The inaugural report on the Global Digital Health Index was subsequently published in 2019 (37). To the best of our knowledge, this inaugural report is the first attempt to evaluate countries' progress in digital health using 19 core indicators grouped under seven categories of key indicators of the WHO/ITU's National eHealth Strategy Toolkit (leadership and governance; strategy and investment; legislation, policy, and compliance; workforce; standards and interoperability; infrastructure; and services and applications). Although the report provided valuable insights into the current state of legislation, policy, and compliance as well as standards and interoperability across countries, only five of 47 countries (Benin, Ethiopia, Mali, Nigeria, Sierra Leone, and Uganda) in the WHO African Region participated in the evaluation. Moreover, the report did not provide any insight on whether countries have standalone regulatory standards and guidance on digital health and whether existing policies and strategies address key regulatory challenges related to the use of health apps for self-management.

For peer review only - http://bmjopen.bmj.com/site/about/guidelines.xhtml

In this paper, we present a protocol for a scoping review to investigate the extent to which regulatory standards and guidance address key regulatory challenges affecting the use of health apps for self-management in countries[1] within the WHO African Region.

Aim and Objectives

Aim

To identify and assess the extent to which regulatory standards and guidance regulate the use of health apps for self-management, and to map out the key stakeholders and their responsibilities in regulating the use of health apps for patient self-management within the WHO African Region countries.

Objectives

The objectives of this review are to:

1. *Identify* regulatory standards and guidance that are available for regulating the use of health apps for self-management within the WHO African Region countries.
2. *Assess* the extent to which the regulatory standards and guidance regulate the use of health apps for self-management in terms of what aspects are regulated, why, how and for whom, and what aspects are not regulated.
3. *Map* out the key stakeholders and their roles in regulating the use of health apps for patient self-management.

Methods

Study design

A team comprising experts and researchers with experience and interest in the relevant disciplines (digital health, self-management, health policy and planning, and scoping review methodology) will design, conduct and report the scoping review. The process of the scoping review will follow the methodological framework for conducting a scoping study originally described by Arksey and O'Malley (2005), and the updated methodological guidance for

[1] Algeria, Angola, Benin, Botswana, Burkina Faso, Burundi, Cameroon, Cape Verde, Central African Republic, Chad, Comoros, Ivory Coast, Democratic Republic of the Congo, Equatorial Guinea, Eritrea, Ethiopia, Gabon, Gambia, Ghana, Guinea, Guinea-Bissau, Kenya, Lesotho, Liberia, Madagascar, Malawi, Mali, Mauritania, Mauritius, Mozambique, Namibia, Niger, Nigeria, Republic of the Congo, Rwanda, São Tomé and Príncipe, Senegal, Seychelles, Sierra Leone, South Africa, South Sudan, Eswatini, Togo, Uganda, Tanzania, Zambia, Zimbabwe.

For peer review only - http://bmjopen.bmj.com/site/about/guidelines.xhtml

conducting a JBI scoping review (39–42). While stage 1 of the framework will guide the formation of the review question and search strategy, stage 2 and stage 3 will subsequently guide the identification of relevant studies and the study selection process respectively. Stage 4 will guide the development of the data-charting form as well as the data charting (extraction) process itself, whereas stage 5 will guide the process of collation, summarisation, and reporting of results. The final stage (stage 6) is optional, and it is meant to guide the process of consultation with stakeholders (if required). The reporting of the scoping review will be guided by the Preferred Reporting Items for Systematic Reviews and Meta-Analyses extension for Scoping Reviews (PRISMA-ScR) checklist (43). The PRISMA-ScR checklist will be completed and attached as an additional file.

This review is not registered with PROSPERO or any similar registration platform as it is not a requirement for scoping reviews to be pre-registered (42).

Stage 1: Identifying the research questions

Research questions

The scoping review will attempt to answer the following research questions, which were generated based on gaps identified from preliminary literature review:

1. What regulatory standards and guidance are available for regulating the use of health apps for self-management within the WHO African Region countries?
2. To what extent do regulatory standards and guidance regulate the use of health apps for self-management in terms of what aspects are regulated, why, how and for whom, and what aspects are not regulated?
3. Who are the key stakeholders and what are their roles in regulating the use of health apps for patient self-management?

Stage 2: Identifying relevant documents

A comprehensive search strategy will be designed by two reviewers with the assistance of a librarian and in consultation with other research team members. The following key terms will be included: policy, legislation, strategy, regulation, standard, criterion, framework, guidance, guideline, digital health, eHealth, app, WHO African Region, and sub-Saharan Africa, and list of all countries within the WHO African Region. We will use truncation to increase yield of results.

For peer review only - http://bmjopen.bmj.com/site/about/guidelines.xhtml

The search strategy will then be used to search for all eligible documents and policy related sources of evidence including conference proceedings and grey literature (committee reports and government reports). Boolean terms (mainly 'OR' and 'AND') will be used to combine search results. 'OR' will be used to combine results within the same category while 'AND' will be used to combine result between different categories to narrow down the search results.

Considering that regulatory standards and guidance are unlikely to be available in scientific databases, we will focus our search on Scopus, Google, OPENGREY, WHO Regional Office for Africa Library (AFROLIB), African Index Medicus (AIM), websites of WHO, ITU and Ministries of Health, and repositories for digital health policies (including ICT Works, WHO directory of eHealth policies and HIS Strengthening Resource Centre). In addition, we will search the reference lists of included documents, and contact key stakeholders in the region, including persons working at the Ministry of Health (or other relevant departments and institutions), WHO Country Offices, and the WHO Regional Office for Africa.

A sample search strategy for Scopus is provided in supplementary file 1.

Stage 3: Study selection

Standalone regulatory standards and guidance; national policies and strategies; and other sources of evidence including committee or government reports (published or unpublished) identified from searches and key contact persons will be imported into Mendeley reference management software to remove duplicates. After removing duplicates, the remaining documents will be imported into Covidence (a web-based tool that allows two reviewers to independently manage article screening and data extraction). Using the predefined eligibility criteria outlined below, two reviewers, working independently, will screen each source of evidence in two stages (title and abstract, and full text) for possible inclusion in the review. Any discrepancies will be discussed in order to reach a consensus. Unresolved disagreements will be further deliberated on and resolved in a steering group meeting involving a third reviewer.

Criteria for selection of documents for this review

Inclusion criteria

The following categories of documents that will be considered for inclusion in this review:

- Standalone regulatory standards and guidance that potentially regulate or provide guidance for the use of health apps.

For peer review only - http://bmjopen.bmj.com/site/about/guidelines.xhtml

- National policies and strategies on digital health developed and produced by countries within the WHO African Region.
- Other sources of evidence (published or unpublished) including committee or government reports that potentially address regulatory issues related to the use of health apps in any WHO African Region country.

Exclusion criteria

Documents that fall within the following categories will be excluded:

- Regulatory standards, guidance, policies, strategies, and committee or government reports that address regulatory issues related to the use of health apps for patient self-management in countries outside the WHO African Region.
- Documents that were written or produced earlier than 2005. This is because it was only in the year 2005 that global efforts toward promoting standards to minimise variability and potential harms that could arise from poorly regulated use of digital health began (25).

Stage 4: Charting (extraction) the data

Two reviewers in consultation with the other members of the research team will develop the data extraction form using an iterative process. At the initial stage of this process, a pilot data extraction will be carried out on at least 10 percent of included sources of evidence. The form will continuously be refined until a consensus is reached.

Following the development of the data extraction form, full data extraction will then be done by the two reviewers independently and any disagreement will be resolved by discussion. Inconsistencies or unresolved issues will be discussed and resolved with a third reviewer in a steering group meeting. Any missing information that is relevant to this review will be sought from the relevant stakeholders.

As recommended by Peters et al. (2020) (42), the data to be extracted will include author (s); year of production or publication; country (where the document was produced or published); type of document (e.g., standalone regulatory standard/guidance, national policy, other sources of evidence including government reports); document development approach (e.g. public-private/multisectoral approach or private sector approach); the aspects of health apps for self-management that are regulated; the reasons why they are regulated; how the regulations are enforced; for which population are the regulations targeted (e.g., apps users, app developers

For peer review only - http://bmjopen.bmj.com/site/about/guidelines.xhtml

and healthcare providers); and the aspects of health apps for self-management that are not regulated; and the key stakeholders and their responsibilities in regulating the use of health apps for patient self-management.

Stage 5: Collating, summarizing, and reporting the results

Unlike systematic reviews, the goal of scoping reviews is not to synthesise the results or outcomes of included sources of evidence. However, as recommended by Peters et al. (2020), the scoping review authors may choose to extract results and descriptively (rather than analytically) map them (42). Therefore, data extracted in stage 4 will be reported using descriptive qualitative content analysis based on the review objectives and as an attempt answer the research questions. The policy analysis framework by Walt and Gilson (1994) (44) will be used to organise findings according to the aspects of the use of health apps for self-management that are regulated, the reasons why they are regulated, how the regulations are enforced, for which population are the regulations targeted. This will be done based on four priori categories, namely: content, context, process, and actors. The Reporting Items for Stakeholder Analysis (i.e., the RISA tool) (45) will be used to guide the mapping of key stakeholders and their responsibilities in regulating the use of health apps for patient self-management.

Following descriptive qualitative content analysis, summary of the key findings will be presented using tables, charts, and geospatial map(s) to aid visualisation (46). More specifically, geospatial mapping will help highlight gaps in coverage of regulatory standards and guidance for regulating the use of health apps for self-management across countries within the WHO African Region.

Stage 6: Consultation

Stage 6 (consultation) is an optional but important component of scoping study methodology (41). While this study does not include stage 6, there are future plans for consultation with patients, healthcare professionals and policy makers in form of interviews, focus group discussions and stakeholders' workshop.

For peer review only - http://bmjopen.bmj.com/site/about/guidelines.xhtml

Patient and Public Involvement

There was no direct involvement of patients and the public in the design of this study. Nevertheless, the interests of patients and the public were among the major factors that informed the study design.

Ethics and Dissemination

This study is mainly a review with no intention to collect primary data. Hence, application for ethical approval is not required.

On completion of the study, a manuscript will be submitted to a peer-reviewed journal for publication while summary of the findings will be shared with relevant organizations. Key findings will be summarised and presented at national, regional, and international conferences.

Acknowledgments

We appreciate Rebecca Jones, the Library Manager and Liaison Librarian at the Charing Cross Library, Imperial College London, for her advice and assistance with the search strategy for the main review.

Footnotes

Contributors

BAB and JC conceived the study. BAB designed the study with contributions from JC and NM. BAB drafted the manuscript and JC, NM, AM, SI, KPF, BIH, and NU read and contributed to it. AM is the clinical lead while JC acts as guarantor for the study. The final manuscript was read and approved by all the authors.

Funding

This work is part of BAB's PhD research which is sponsored by the Nigerian government. AM and JC are supported by NIHR Applied Research Collaboration North-West London, grant number NIHR200180. The views expressed in this publication are those of the authors and not necessarily those of the Nigerian government or the NIHR or the Department of Health and Social Care.

For peer review only - http://bmjopen.bmj.com/site/about/guidelines.xhtml

Competing interests

None declared.

Patient consent for publication

Not required

For peer review only

For peer review only - http://bmjopen.bmj.com/site/about/guidelines.xhtml

References

1. Lorig KR, Holman HR. Self-management education: History, definition, outcomes, and mechanisms. Annals of Behavioral Medicine [Internet]. 2003 Aug 1 [cited 2021 Sep 29];26(1):1–7. Available from: https://academic.oup.com/abm/article/26/1/1/4630312

2. Barlow J, Wright C, Sheasby J, Turner A, Hainsworth J. Self-management approaches for people with chronic conditions: a review. Patient Education and Counseling. 2002 Oct 1;48(2):177–87.

3. Chaney SC, Mechael P, Self-Care Trailblazer Group. Digital Self Care: A Framework for Design, Implementation & Evaluation [Internet]. 2020 [cited 2021 May 20]. Available from: https://www.psi.org/wp-content/uploads/2020/09/Digital-Self-Care.pdf

4. Aikens JE, Zivin K, Trivedi R, Piette JD. Diabetes self-management support using mHealth and enhanced informal caregiving. Journal of Diabetes and its Complications [Internet]. 2014;28(2):171–6. Available from: https://www.sciencedirect.com/science/article/pii/S1056872713002869

5. Jeffrey B, Bagala M, Creighton A, Leavey T, Nicholls S, Wood C, et al. Mobile phone applications and their use in the self-management of Type 2 Diabetes Mellitus: a qualitative study among app users and non-app users. Diabetol Metab Syndr [Internet]. 2019 [cited 2021 Jun 8];11:84. Available from: https://doi.org/10.1186/s13098-019-0480-4

6. RiskIQ. Mobile App Threat Landscape Report [Internet]. 2020 [cited 2021 Jul 19]. Available from: https://www.riskiq.com/2020-mobile-threat-landscape-report-thank-you/

7. Bene BA, O'Connor S, Mastellos N, Majeed A, Fadahunsi KP, O'Donoghue J. Impact of mobile health applications on self-management in patients with type 2 diabetes mellitus: protocol of a systematic review. BMJ Open [Internet]. 2019 Jun 1 [cited 2021 Jul 19];9(6):e025714. Available from: https://bmjopen.bmj.com/content/9/6/e025714

8. Peng W, Kanthawala S, Yuan S, Hussain SA. A qualitative study of user perceptions of mobile health apps. BMC Public Health [Internet]. 2016 Nov 14 [cited 2021 Sep 20];16(1):1–11. Available from: https://bmcpublichealth.biomedcentral.com/articles/10.1186/s12889-016-3808-0

9. Krebs P, Duncan DT. Health App Use Among US Mobile Phone Owners: A National Survey. JMIR mHealth and uHealth [Internet]. 2015 Nov 4 [cited 2021 Sep 20];3(4):e4924. Available from: https://mhealth.jmir.org/2015/4/e101

10. Anderson K, Burford O, Emmerton L. Mobile Health Apps to Facilitate Self-Care: A Qualitative Study of User Experiences. PLOS ONE [Internet]. 2016 May 1 [cited 2021 Sep 20];11(5):e0156164. Available from: https://journals.plos.org/plosone/article?id=10.1371/journal.pone.0156164

11. Gordon WJ, Landman A, Zhang H, Bates DW. Beyond validation: getting health apps into clinical practice. npj Digital Medicine 2020 3:1 [Internet]. 2020 Feb 3 [cited 2021 Sep 20];3(1):1–6. Available from: https://www.nature.com/articles/s41746-019-0212-z

For peer review only

12. Bene B, O'Connor S, Majeed A, O'Donoghue J, Fadahunsi K, Mastellos N. The impact of mobile health applications on self-management in adults with type 2 diabetes mellitus: systematic review and meta-analysis [Internet]. PROSPERO CRD42017071106. 2017 [cited 2021 Sep 30]. Available from: https://www.crd.york.ac.uk/prospero/display_record.php?ID=CRD42017071106

13. Aitken M, Lyle J. Patient Adoption of mHealth: Use, Evidence and Remaining Barriers to Mainstream Acceptance [Internet]. 2015 [cited 2021 May 21]. Available from: www.theimsinstitute.org

14. Olu O, Muneene D, Bataringaya JE, Nahimana MR, Ba H, Turgeon Y, et al. How Can Digital Health Technologies Contribute to Sustainable Attainment of Universal Health Coverage in Africa? A Perspective. Frontiers in Public Health. 2019 Nov 15;7.

15. Wanless D. Securing our Future Health: Taking a Long-Term View Final Report. 2002.

16. WHO. The MAPS Toolkit: mHealth Assessment and Planning for Scale [Internet]. Geneva; 2015 [cited 2021 Mar 15]. Available from: www.who.int

17. Wallis L, Blessing P, Dalwai M, Shin S do. Integrating mHealth at point of care in low- and middle-income settings: the system perspective. Global Health Action [Internet]. 2017 [cited 2021 Jul 18];10(sup3). Available from: /pmc/articles/PMC5645717/

18. Piette JD, Lun KC, Moura LA, Fraser HS, Mechael PN, Powell J, et al. Policy & practice Impacts of e-health on the outcomes of care in low-and middle-income countries: where do we go from here? Bull World Health Organ. 2012;90:365–72.

19. Mechael P, Batavia H, Kaonga N, Searle S, Kwan A, Goldberger A, et al. Barriers and Gaps Affecting mHealth in Low and Middle Income Countries: Policy White Paper [Internet]. Columbia; 2010. Available from: http://www.globalproblems-globalsolutions-files.org/pdfs/mHealth_Barriers_White_Paper.pdf

20. World Health Organization. Global diffusion of eHealth: Making universal health coverage achievable [Internet]. Geneva; 2016 [cited 2021 Jul 18]. Available from: http://apps.who.int/bookorders.

21. GSM Association. The Mobile Economy Sub-Saharan Africa 2020 [Internet]. 2020 [cited 2021 Sep 28]. Available from: https://www.gsma.com/mobileeconomy/wp-content/uploads/2020/09/GSMA_MobileEconomy2020_SSA_Eng.pdf

22. Bahia K, Suardi S. The State of Mobile Internet Connectivity 2019 [Internet]. 2019 [cited 2021 Sep 27]. Available from: https://www.gsma.com/mobilefordevelopment/wp-content/uploads/2019/07/GSMA-State-of-Mobile-Internet-Connectivity-Report-2019.pdf

23. Aitken M, Gauntlett C. Patient Apps for Improved Healthcare: From Novelty to Mainstream [Internet]. IMS Institute for Healthcare Informatics. Parsippany, NJ; 2013. Available from: http://www.imshealth.com/portal/site/imshealth

24. WHO, ITU. National eHealth Strategy Toolkit [Internet]. 2012 [cited 2021 Jun 28]. Available from: https://www.itu.int/pub/D-STR-E_HEALTH.05-2012

25. WHO. Global strategy on digital health 2020-2025 [Internet]. 2021 [cited 2021 Jun 23]. Available from: http://apps.who.int/bookorders.

For peer review only - http://bmjopen.bmj.com/site/about/guidelines.xhtml

26. ORCHA. What are ORCHA's Best Diabetes Apps? [Internet]. 2019 [cited 2021 Jul 19]. Available from: https://orchahealth.com/best-diabetes-apps/

27. ORCHA. Coronavirus: Apps to help the elderly [Internet]. 2020 [cited 2021 Jul 19]. Available from: https://orchahealth.com/coronavirus-apps-to-help-the-elderly/

28. Huckvale K, Adomaviciute S, Prieto JT, Leow MK, Car J. Smartphone apps for calculating insulin dose: a systematic assessment. BMC Medicine [Internet]. 2015;13(1):106. Available from: http://www.ncbi.nlm.nih.gov/pubmed/25943590

29. Huckvale K, Car M, Morrison C, Car J. Apps for asthma self-management: a systematic assessment of content and tools. BMC medicine [Internet]. 2012;10(1):144. Available from: http://www.biomedcentral.com/1741-7015/10/144

30. Huckvale K, Morrison C, Ouyang J, Ghaghda A, Car J. The evolution of mobile apps for asthma: an updated systematic assessment of content and tools. BMC Medicine [Internet]. 2015;13(1):58. Available from: http://www.ncbi.nlm.nih.gov/pubmed/25857569

31. Huckvale K, Prieto JT, Tilney M, Benghozi P-J, Car J. Unaddressed privacy risks in accredited health and wellness apps: a cross-sectional systematic assessment. BMC Medicine 2015 13:1 [Internet]. 2015 Sep 25 [cited 2021 Jul 21];13(1):1–13. Available from: https://bmcmedicine.biomedcentral.com/articles/10.1186/s12916-015-0444-y

32. Huckvale K, Torous J, Larsen ME. Assessment of the Data Sharing and Privacy Practices of Smartphone Apps for Depression and Smoking Cessation. JAMA Network Open [Internet]. 2019 Apr 5 [cited 2021 Jul 21];2(4):e192542–e192542. Available from: https://jamanetwork.com/journals/jamanetworkopen/fullarticle/2730782

33. Unsworth H, Dillon B, Collinson L, Powell H, Salmon M, Oladapo T, et al. The NICE Evidence Standards Framework for digital health and care technologies – Developing and maintaining an innovative evidence framework with global impact: https://doi.org/101177/20552076211018617 [Internet]. 2021 Jun 24 [cited 2021 Jul 28];7:205520762110186. Available from: https://journals.sagepub.com/doi/full/10.1177/20552076211018617

34. Wachter RM. Making IT Work: Harnessing the Power of Health Information Technology to Improve Care in England [Internet]. 2016 [cited 2021 Jul 22]. Available from: https://assets.publishing.service.gov.uk/government/uploads/system/uploads/attachment_data/file/550866/Wachter_Review_Accessible.pdf

35. WHO, ITU. Digital Health Platform Handbook: Building a Digital Information Infrastructure (Infostructure) for Health. Geneva; 2020.

36. Ibeneme S, Ukor N, Ongom M, Dasa T, Muneene D, Okeibunor J. Strengthening capacities among digital health leaders for the development and implementation of national digital health programs in Nigeria. In: BMC Proceedings [Internet]. BioMed Central; 2020 [cited 2021 May 23]. p. 1–12. Available from: https://doi.org/10.1186/s12919-020-00193-1

37. Mechael P, Edelman JK. Global Digital Health Index: The State of Digital Health 2019. 2019.

38. Global Digital Health Index [Internet]. [cited 2021 Jul 29]. Available from: https://www.digitalhealthindex.org/

39. Arksey H, O'Malley L. Scoping studies: Towards a methodological framework. International Journal of Social Research Methodology: Theory and Practice. 2005 Feb;8(1):19–32.

For peer review only

40. Anderson S, Allen P, Peckham S, Goodwin N. Asking the right questions: Scoping studies in the commissioning of research on the organization and delivery of health services. Vol. 6, Health Research Policy and Systems. 2008.

41. Levac D, Colquhoun H, O'Brien KK. Scoping studies: Advancing the methodology. Implementation Science. 2010 Sep 20;5(1).

42. Peters M, Godfrey C, McInerney P, Munn Z, Trico A, Khalil H. Scoping Reviews. In: Aromataris E, Munn Z, editors. JBI Manual for Evidence Synthesis [Internet]. JBI; 2020 [cited 2021 Aug 23]. Available from: https://synthesismanual.jbi.global. https://doi.org/10.46658/JBIMES-20-12

43. Tricco AC, Lillie E, Zarin W, O'Brien KK, Colquhoun H, Levac D, et al. PRISMA Extension for Scoping Reviews (PRISMA-ScR): Checklist and Explanation. https://doi.org/107326/M18-0850 [Internet]. 2018 Sep 4 [cited 2021 Jul 30];169(7):467–73. Available from: https://www.acpjournals.org/doi/abs/10.7326/M18-0850

44. Walt G, Gilson L. Reforming the health sector in developing countries: the central role of policy analysis [Internet]. Vol. 9, HEALTH POLICY AND PLANNING. 1994. Available from: https://academic.oup.com/heapol/article/9/4/353/649125

45. Franco-Trigo L, Fernandez-Llimos F, Martínez-Martínez F, Benrimoj SI, Sabater-Hernández D. Stakeholder analysis in health innovation planning processes: A systematic scoping review. Health Policy [Internet]. 2020 [cited 2021 Sep 3];124:1083–99. Available from: https://doi.org/10.1016/j.healthpol.2020.06.012

46. Wariri O, Okomo U, Kwarshak YK, Murray KA, Grundy C, Kampmann B. Timeliness of routine childhood vaccination in low- and middle-income countries, 1978–2021: Protocol for a scoping review to map methodologic gaps and determinants. PLOS ONE [Internet]. 2021 Jun 1 [cited 2021 Aug 22];16(6):e0253423. Available from: https://journals.plos.org/plosone/article?id=10.1371/journal.pone.0253423

For peer review only

For peer review only - http://bmjopen.bmj.com/site/about/guidelines.xhtml

Supplementary file 2: Preferred Reporting Items for Systematic reviews and Meta-Analyses extension for Scoping Reviews (PRISMA-ScR) Checklist

SECTION	ITEM	PRISMA-ScR CHECKLIST ITEM	REPORTED ON PAGE #
**TITLE**			
Title	1	Identify the report as a scoping review.	Front page
**ABSTRACT**			
Structured summary	2	Provide a structured summary that includes (as applicable): background, objectives, eligibility criteria, sources of evidence, charting methods, results, and conclusions that relate to the review questions and objectives.	i
**INTRODUCTION**			
Rationale	3	Describe the rationale for the review in the context of what is already known. Explain why the review questions/objectives lend themselves to a scoping review approach.	1-3
Objectives	4	Provide an explicit statement of the questions and objectives being addressed with reference to their key elements (e.g., population or participants, concepts, and context) or other relevant key elements used to conceptualize the review questions and/or objectives.	3
**METHODS**			
Protocol and registration	5	Indicate whether a review protocol exists; state if and where it can be accessed (e.g., a Web address); and if available, provide registration information, including the registration number.	N/A
Eligibility criteria	6	Specify characteristics of the sources of evidence used as eligibility criteria (e.g., years considered, language, and publication status), and provide a rationale.	5-6
Information sources*	7	Describe all information sources in the search (e.g., databases with dates of coverage and contact with authors to identify additional sources), as well as the date the most recent search was executed.	4-5
Search	8	Present the full electronic search strategy for at least 1 database, including any limits used, such that it could be repeated.	Supplementary file 1
Selection of sources of evidence†	9	State the process for selecting sources of evidence (i.e., screening and eligibility) included in the scoping review.	5-6
Data charting process‡	10	Describe the methods of charting data from the included sources of evidence (e.g., calibrated forms or forms that have been tested by the team before their use, and whether data charting was done independently or in duplicate) and any processes for obtaining and confirming data from investigators.	6
Data items	11	List and define all variables for which data were sought and any assumptions and simplifications made.	N/A
Critical appraisal of individual	12	If done, provide a rationale for conducting a critical appraisal of included sources of evidence; describe	N/A

For peer review only

SECTION	ITEM	PRISMA-ScR CHECKLIST ITEM	REPORTED ON PAGE #
sources of evidence§		the methods used and how this information was used in any data synthesis (if appropriate).	
Synthesis of results	13	Describe the methods of handling and summarizing the data that were charted.	N/A
**RESULTS**			
Selection of sources of evidence	14	Give numbers of sources of evidence screened, assessed for eligibility, and included in the review, with reasons for exclusions at each stage, ideally using a flow diagram.	N/A
Characteristics of sources of evidence	15	For each source of evidence, present characteristics for which data were charted and provide the citations.	N/A
Critical appraisal within sources of evidence	16	If done, present data on critical appraisal of included sources of evidence (see item 12).	N/A
Results of individual sources of evidence	17	For each included source of evidence, present the relevant data that were charted that relate to the review questions and objectives.	N/A
Synthesis of results	18	Summarize and/or present the charting results as they relate to the review questions and objectives.	N/A
**DISCUSSION**			
Summary of evidence	19	Summarize the main results (including an overview of concepts, themes, and types of evidence available), link to the review questions and objectives, and consider the relevance to key groups.	N/A
Limitations	20	Discuss the limitations of the scoping review process.	N/A
Conclusions	21	Provide a general interpretation of the results with respect to the review questions and objectives, as well as potential implications and/or next steps.	N/A
**FUNDING**			
Funding	22	Describe sources of funding for the included sources of evidence, as well as sources of funding for the scoping review. Describe the role of the funders of the scoping review.	8

JBI = Joanna Briggs Institute; PRISMA-ScR = Preferred Reporting Items for Systematic reviews and Meta-Analyses extension for Scoping Reviews.
* Where *sources of evidence* (see second footnote) are compiled from, such as bibliographic databases, social media platforms, and Web sites.
† A more inclusive/heterogeneous term used to account for the different types of evidence or data sources (e.g., quantitative and/or qualitative research, expert opinion, and policy documents) that may be eligible in a scoping review as opposed to only studies. This is not to be confused with *information sources* (see first footnote).
‡ The frameworks by Arksey and O'Malley (6) and Levac and colleagues (7) and the JBI guidance (4, 5) refer to the process of data extraction in a scoping review as data charting.
§ The process of systematically examining research evidence to assess its validity, results, and relevance before using it to inform a decision. This term is used for items 12 and 19 instead of "risk of bias" (which is more applicable to systematic reviews of interventions) to include and acknowledge the various sources of evidence that may be used in a scoping review (e.g., quantitative and/or qualitative research, expert opinion, and policy document).

From: Tricco AC, Lillie E, Zarin W, O'Brien KK, Colquhoun H, Levac D, et al. PRISMA Extension for Scoping Reviews (PRISMAScR): Checklist and Explanation. Ann Intern Med. 2018;169:467–473. doi: 10.7326/M18-0850.

For peer review only - http://bmjopen.bmj.com/site/about/guidelines.xhtml

Supplementary file 1: Sample search strategy for Scopus

Database(s): **Scopus** 1960 to October 2021 (final search limited to 2005 – current)

Search Strategy:

ID	Query	Documents
#6	((polic* OR legislat* OR strateg* OR regulat* OR standard* OR criter* OR framework* OR guideline*)) AND (digital AND health OR ehealth OR e-health OR mhealth OR m-health) AND ((application AND software OR app* OR software AND app* OR mobile AND app* OR mobile AND phone AND app* OR medical AND app*)) AND ((world AND health AND organization OR who AND african AND region OR africa OR sub-saharan AND africa OR "Africa South of the Sahara") OR (algeria OR angola OR benin OR botswana OR burkina AND faso OR burundi OR cameroon OR cape AND verde OR cabo AND verde OR central AND african AND republic OR chad OR comoros OR ivory AND coast OR cote AND d'ivoire OR democratic AND republic AND of AND the AND congo OR equatorial AND guinea OR eritrea OR ethiopia OR gabon OR gambia OR ghana OR guinea OR guinea-bissau OR kenya OR lesotho OR liberia OR madagascar OR malawi OR mali OR mauritania OR mauritius OR mozambique OR namibia OR niger OR nigeria OR republic AND of AND the AND congo OR congo OR rwanda OR são AND tomé AND príncipe OR saint AND thomas AND prince OR senegal OR seychelles OR sierra AND leone OR south AND africa OR south AND sudan OR eswatini OR togo OR uganda OR tanzania OR zambia OR zimbabwe)) AND (LIMIT-TO (PUBYEAR , 2021) OR LIMIT-TO (PUBYEAR , 2020) OR LIMIT-TO (PUBYEAR , 2019) OR LIMIT-TO (PUBYEAR , 2018) OR LIMIT-TO (PUBYEAR , 2017) OR LIMIT-TO (PUBYEAR , 2016) OR LIMIT-TO (PUBYEAR , 2015) OR LIMIT-TO (PUBYEAR , 2014) OR LIMIT-TO (PUBYEAR , 2013) OR LIMIT-TO (PUBYEAR , 2012) OR LIMIT-TO (PUBYEAR , 2011) OR LIMIT-TO (PUBYEAR , 2010) OR LIMIT-TO (PUBYEAR , 2009) OR LIMIT-TO (PUBYEAR , 2008) OR LIMIT-TO (PUBYEAR , 2007) OR LIMIT-TO (PUBYEAR , 2006) OR LIMIT-TO (PUBYEAR , 2005))	1,054
#5	((polic* OR legislat* OR strateg* OR regulat* OR standard* OR criter* OR framework* OR guideline*)) AND (digital AND health OR ehealth OR e-health OR mhealth OR m-health) AND ((application AND software OR app* OR software AND app* OR mobile AND app* OR mobile AND phone AND app*	1,057

For peer review only

	OR medical AND app*)) AND ((world AND health AND organization OR who AND african AND region OR africa OR sub-saharan AND africa OR "Africa South of the Sahara") OR (algeria OR angola OR benin OR botswana OR burkina AND faso OR burundi OR cameroon OR cape AND verde OR cabo AND verde OR central AND african AND republic OR chad OR comoros OR ivory AND coast OR cote AND d'ivoire OR democratic AND republic AND of AND the AND congo OR equatorial AND guinea OR eritrea OR ethiopia OR gabon OR gambia OR ghana OR guinea OR guinea-bissau OR kenya OR lesotho OR liberia OR madagascar OR malawi OR mali OR mauritania OR mauritius OR mozambique OR namibia OR niger OR nigeria OR republic AND of AND the AND congo OR congo OR rwanda OR são AND tomé AND príncipe OR saint AND thomas AND prince OR senegal OR seychelles OR sierra AND leone OR south AND africa OR south AND sudan OR eswatini OR togo OR uganda OR tanzania OR zambia OR zimbabwe))	
#4	(world AND health AND organization OR who AND african AND region OR africa OR sub-saharan AND africa OR "Africa South of the Sahara") OR (algeria OR angola OR benin OR botswana OR burkina AND faso OR burundi OR cameroon OR cape AND verde OR cabo AND verde OR central AND african AND republic OR chad OR comoros OR ivory AND coast OR cote AND d'ivoire OR democratic AND republic AND of AND the AND congo OR equatorial AND guinea OR eritrea OR ethiopia OR gabon OR gambia OR ghana OR guinea OR guinea-bissau OR kenya OR lesotho OR liberia OR madagascar OR malawi OR mali OR mauritania OR mauritius OR mozambique OR namibia OR niger OR nigeria OR republic AND of AND the AND congo OR congo OR rwanda OR são AND tomé AND príncipe OR saint AND thomas AND prince OR senegal OR seychelles OR sierra AND leone OR south AND africa OR south AND sudan OR eswatini OR togo OR uganda OR tanzania OR zambia OR zimbabwe)	143,892
#3	(application AND software OR app* OR software AND app* OR mobile AND app* OR mobile AND phone AND app* OR medical AND app*)	208,315
#2	digital AND health OR ehealth OR e-health OR mhealth OR m-health	495,739
#1	(polic* OR legislat* OR strateg* OR regulat* OR standard* OR criter* OR framework* OR guideline*)	30,603,666

For peer review only
